# Regime Shift in Sandy Beach Microbial Communities following *Deepwater Horizon* Oil Spill Remediation Efforts

**DOI:** 10.1371/journal.pone.0102934

**Published:** 2014-07-18

**Authors:** Annette Summers Engel, Axita A. Gupta

**Affiliations:** 1 Department of Geology and Geophysics, Louisiana State University, Baton Rouge, Louisiana, United States of America; 2 Department of Earth and Planetary Sciences, University of Tennessee, Knoxville, Tennessee, United States of America; U.S. Geological Survey, United States of America

## Abstract

Sandy beaches support a wide variety of underappreciated biodiversity that is critical to coastal ecosystems. Prior to the 2010 *Deepwater Horizon* oil spill, the diversity and function of supratidal beach sediment microbial communities along Gulf of Mexico coastlines were not well understood. As such, it was unclear if microbial community compositional changes would occur following exposure to beached oil, if indigenous communities could biodegrade oil, or how cleanup efforts, such as sand washing and sediment redistribution, would impact microbial ecosystem resiliency. Transects perpendicular to the shoreline were sampled from public beaches on Grand Isle, Louisiana, and Dauphin Island, Alabama, over one year. Prior to oil coming onshore, elevated levels of bacteria associated with fecal contamination were detected (e.g., Enterobacteriales and Campylobacterales). Over time, significant shifts within major phyla were identified (e.g., Proteobacteria, Firmicutes, Actinobacteria) and fecal indicator groups were replaced by taxa affiliated with open-ocean and marine systems (e.g., Oceanospirillales, Rhodospirillales, and Rhodobacterales). These new bacterial groups included putative hydrocarbon degraders, similar to those identified near the oil plume offshore. Shifts in the microbial community composition strongly correlated to more poorly sorted sediment and grain size distributional changes. Natural oceanographic processes could not account for the disrupted sediment, especially from the backshore well above the maximum high-tide levels recorded at these sites. Sand washing and tilling occurred on both open beaches from August through at least December 2010, which were mechanisms that could replace fecal indicator groups with open-ocean groups. Consequently, remediation efforts meant to return beaches to pre-spill compositions caused a regime shift that may have added potential ecosystem function, like hydrocarbon degradation, to the sediment. Future research will need to assess the persistence and impact of the newly formed microbial communities to the overall sandy beach ecosystems.

## Introduction

Sandy beaches are highly dynamic coastal buffer zones where the atmosphere, continents, and the oceans interact. A sandy beach includes the geological area parallel to the shoreline that gently slopes from the supratidal, backshore dune with permanent vegetation to the subtidal swash zone at the ocean-water interface, usually at the mean low-tide line, where unconsolidated sediments are deposited and reworked by the highly energetic open ocean [1]. Sandy beaches dominate the world’s open coastlines by covering about 70% of continental shelves [2]. They comprise an important part of any littoral or coastal ecosystem with a wide range of under-appreciated biodiversity [2], and are valued for recreational tourism and fishing economies [3], [4]. Subtidal sands filter and accumulate both organic and inorganic materials [5], [6] and have diverse microbial communities that can mineralize up to 70% of organic matter reaching the beach (e.g., [7], [8]). In contrast, supratidal sands generally have low organic matter content and concentrations of other reactive substances that could support biological processes (e.g., [9]). Accordingly, most microbiology research has focused at the subtidal zone [8], [10]–[15]. But, the microbial diversity and associated processes from supratidal sandy beaches, including dunes, foredunes, exposed open beach slopes, or back-beach berms, have the potential to impact hydrological, sedimentological, and biogeochemical processes that occur throughout the whole beach system.

The dearth of microbiology research from Gulf of Mexico supratidal sandy beaches was acutely realized at the time of the Macondo prospect well failure caused by the *Deepwater Horizon* explosion in April 2010. Model projections done by the National Oceanic and Atmospheric Administration (NOAA) based on historical wind and ocean currents indicated 80 to 100% probability of surface oil making landfall, and a ∼40–60% chance that oil and dispersants would affect a significant portion of the Gulf coast [16]. By July 2010, the mapped and confirmed extent of oil contamination on shorelines, determined by NASA/MODIS and NOAA’s Shoreline Cleanup and Assessment Technique (SCAT) mapping [17], included well-known, economically important public sandy beaches of Louisiana, Mississippi, Alabama, and Florida [18]. Because of the challenge to estimate natural biodegradation times, beached oil and tar balls (also referred to as surface residue balls, [18]) were removed manually. For heavily contaminated shorelines, remediation intensity was greater, with sand being washed on the beach or removed completely using large machines and replaced with sediment from other parts of the beach [18], [19]. Recreational beaches, even if only moderately to barely contaminated, were also intensely remediated to reach “2010 No Further Treatment” guidelines [18].

Our study evaluated supratidal beach microbial community compositions and potential controls on diversity that could be used to determine microbial ecosystem resiliency following disturbance. We defined a disturbance as a chemical or physical condition beyond its normal dynamic range that would induce a response to the biological system [20]–[23]. Ecosystem resiliency was defined as the amount of time to return communities to pre-disturbance conditions (sensu stricto, [22]). We considered that the type, extent, and duration of predominately mechanical or physical remediation technologies on the beaches [19], [24] would impact community composition, regardless of the potential level of oiling on the beaches. We focused on two public beaches on Grand Isle, Louisiana, and Dauphin Island, Alabama (Figure S1). Both beaches were considered at risk for adverse economic and ecological impacts, as well as were understudied with respect to their supratidal beach microbiology. We assumed that the beaches would receive attentive and potentially intensive physical remediation as part of the oil spill emergency response to maintain recreational and economic interests [25]. Moreover, both beaches are part of the United States Environmental Protection Agency (US EPA) Beaches Environmental Assessment and Coastal Health (BEACH) Act [26], [27] monitoring program, which meant that the recreational waters must meet water quality standards for fecal contamination based on bacterial indicator and pathogenic species (e.g., *E. coli* and enterococci). This monitoring program information provided a way to assess beach water quality changes over time. Our sampling efforts started early May 2010, less than one month after the *Deepwater Horizon* explosion and before oil reached the beaches, which distinguishes our research from the later collections on other Gulf Coast sandy beaches [15], [28]. Overall, the results provide important information about sandy beach microbial ecosystem dynamics from analyses of 16S rRNA 454 tag pyrosequences. Based on physical and chemical characterization of beach sediments, we identified significant microbial community regime shifts [29] from areas of the supratidal beach that were physically impacted by remediation efforts. Pre-spill communities associated with fecal contamination were replaced by communities known from open-ocean marine habitats and affiliated with putative hydrocarbon degradation metabolisms. Regime shifts of this scale may have improved overall beach health, but additional research is needed to understand what consequences there are on overall beach ecosystem dynamics and processes due to shifts in taxonomic and functional microbial diversity.

## Materials and Methods

### Ethics Statement

No specific permits were required for the described field studies because no sample collection was done on protected or privately-owned beaches, and field work did not impact animals.

### Sampling locations

Sediment samples were collected from the dune crest to the swash zone along transects perpendicular to the shoreline. Transect sampling locations included dunes, the back beach slope, and foreshore beach area landward of the beach berm (Figure S2 and Figure S3). Latitude and longitude coordinates are available from Table S1. Beach profiles were roughly mapped to assess large-scale changes in overall beach structure. Our sampling effort started in early May through early June 2010, before oil came onshore at both beaches based on NOAA SCAT maps and reports, available through the Environmental Response Management Application (ERMA) Deepwater Gulf Response web-based GIS tool (http://gomex.erma.noaa.gov/). Levels of oiling were categorized as no to trace oiling, very light to light oiling, and moderate to heavy oiling, as well as light to heavy tar ball coverage [17], [18]. To include the extent of physical remediation on each of the beaches in our statistical data analysis, we assigned values from 0 to 1 to indicate the extent of remediation, with 0 indicating no physical remediation, 0.5 for intermediate efforts like manual tar ball cleanup, and some sifting or raking. A value of 1 was assigned for extensive physical efforts like sand washing and whole-scale beach sand removal.

Grand Isle is one of the Gulf of Mexico’s barrier islands. Sediments along the dissipative beach are fine to very-fine grained quartz and minor feldspar sand with clay [30]. We acquired samples at Grand Isle from the public, unmanaged eastern end of the island, starting on May 2, 2010 (Figure S2). Patchy tar balls and then oil sheets began washing onshore from May 19 [31], [32]. Light to moderate oiling and moderate coverage of tar balls in the swash zone and foreshore followed through May 29, which coincided with sampling on May 22. We noted a laterally continuous perched oily zone approx. 20 cm below the sand surface in the foreshore beach where we sampled. Heavy oiling began June 2 and continued until August 3. We sampled the beach August 15, 2010, and noted tar balls on the beach where we sampled from the swash zone. Moderate to light oiling occurred from August 3 through September 17 based on SCAT maps. During this time period until after January 2011, access to the heavily oiled beach was restricted so that large equipment like sand washers and tillers could mechanically remove oil to “No Further Treatment” conditions [18]. In a study by Allan et al. [16] to investigate polycyclic aromatic hydrocarbon compounds (PAHs) along shorelines impacted by the *Deepwater Horizon* oil spill, elevated levels of PAHs were measured in July 2010 at Grand Isle, but levels decreased by an order of magnitude by August 2010. We visited on December 11, 2010, but were unable to sample because of physical remediation. We resampled the beach May 14, 2011, and moderate to light oiling was reported from SCAT mapping in August 2011. We did not observe oil or tar balls on the beach when we sampled in May 2011.

Dauphin Island is a high-profile barrier island and beaches are also dissipative, with predominately fine- to medium-sized quartz sands [33]–[35]. The vegetated sand dunes on Dauphin Island are among the highest elevation, aggradational and progradational dunes along the Gulf Coast. The dune formed from generally low erosion rates, although sections of the island have recently experienced high rates of coastal beach erosion (e.g., [34], [36]). Sediment samples were collected from the public beach on the south side of the island, on the historic Pelican/Sand Island that previously migrated landward and extended a spit offshore (e.g., [25], [34], [36]). The first report of tar balls was late May 2010, with light and patchy occurrences of tar balls and cakes starting the first week of June 2010 on the Pelican Island beach [31]. We sampled on May 3, May 9, and June 1 (Figure S3), and observed tar balls only on June 1. Initially, tar balls were manually removed by ground crews using shovels or scoops. Light oiling and light tar ball coverage occurred from July 12– August 3, although moderate oiling occurred on other parts of the island according to SCAT reports. We resampled the beach June 14, August 14, and December 10, 2010. By July 2010, tar balls were removed with sand washing machines that could cover a broader area of the beach [19]. Tar balls could be found in December 2010 on the sediment surface, and then as smears of oil residue in thin bands at more than 5 cm depth, and up to 25 cm depth in the swash and beach berm zones after December 2010 through May 2011. We resampled the beach on May 13, 2011. During the year, large piles of sand were moved around on the beach to protect intertidal, dune, and back-dune areas [25]. Sand was taken from the open beach to create the sand piles and new berms, and to fill sand-box booms offshore. Following these activities, back-beach ponds formed on the beach slope that persisted from December 2010 to May 2011. August 2011 SCAT reports noted trace to light oiling on the beach. For this study, attempts were made to have transects as close as possible to earlier sampling times, although the complete restructuring of the beach forced non-overlapping sample transects. None of the sediment samples collected were obviously contaminated by oil. This was similar to findings from Kostka et al. [6] of subtidal beach sands from Pensacola, Florida, that were examined after the *Deepwater Horizon* incident. However, by May 2011, tar balls were still recoverable from the Dauphin Island beaches, and reports indicated that nearby Gulf Shores, Alabama, had elevated levels of PAHs at the shoreline [16].

### Oceanographic and meteorological data

Oceanographic and meteorological data for the sampling locations at Grand Isle and Dauphin Island were acquired from NOAA’s National Data Buoy Center (NDBC) (http://www.ndbc.noaa.gov/maps/west_gulf_hist.shtml). Gage height, ocean temperature, and salinity data were acquired from the United States Geological Survey (USGS) National Water Information System (NWIS) web interface for station #07380251 (29°25′21″ N 89°57′02″ W) (http://waterdata.usgs.gov/nwis/nwisman/?site_no=07380251&agency_cd=USGS). The arithmetic mean of hourly tide heights were collated from mean sea level (MSL) data from the National Ocean Service (NOS) station #8761724 (29° 15.8′ N 89° 57.4′ W) for Grand Isle (http://tidesandcurrents.noaa.gov/geo.shtml?location=8761724). The arithmetic mean of hourly tide heights according to MSL were acquired from the NOAA NOS station #8735180 (30°15′ N 88°4.5′ W) for Dauphin Island (http://tidesandcurrents.noaa.gov/geo.shtml?location=8735180). Data for Dauphin Island from the NDBC station DPIA1 (30°14′54″ N 88°4′24″ W) included wind direction, wind speed, wind gust, barometric pressure, and air temperature. Ocean temperature and salinity were collated from the Dauphin Island Sea Lab, Coastal Marine Station DPHA1 (30°15′5″ N 88°4′40″ W) (http://www.ndbc.noaa.gov/station_page.php?station=dpha1).

### Beach water quality data

Publically available National Resources Defense Council (NRDC) records for beach closures and beach water quality were assessed for Grand Isle and Dauphin Island from 2009–2011 [37]–[39]. Also, for Grand Isle, 2010–2012 swimming seasons were further evaluated from the Louisiana BEACH Grant Reports submitted by the Louisiana Department of Health and Hospitals (LDHH) [40]–[42]. LDHH obtains samples from at least 30 cm below the water surface and in at least ∼1 m of water at the shoreline [43]. Exceedance of the *Enterococcus* geometric mean test for Louisiana state standards is >35 colony-forming-units (CFU)/100 ml for five samples [39]. For Dauphin Island, the 2009–2011 swimming seasons were also evaluated from US EPA summary reports [44]–[46]. In Alabama, a single water sample, also taken below the surface and in 1 m water depth, needs to have more than 10^4^ CFU/100 ml enterococci to be issued an advisory, but the state does not order beach closures [37].

### Sediment sampling and characterization

Sediment samples were aseptically collected in triplicate at three different depths, where possible: surface or 0 cm (denoted as ‘A’); 5 cm below the surface (denoted ‘B’); 20 cm the below surface (denoted ‘C’). All samples were stored at 4°C for transport, then at −20°C until sample analyses. Sediment pH based on the soluble salt content was estimated in triplicate using an Accumet XL125 dual channel pH/ion meter and double-junction electrode (Fisher Scientific) from of 1∶1 mixture of fresh sediment and18 MΩ distilled and deionized water after shaking for 30 min [47]. Total sediment water and organic carbon contents were determined from a modified loss on ignition (LOI) method optimized for the sediment conditions [48]. Briefly, triplicate samples were weighed separately, and all aliquots were incubated at ∼80–90°C for up to 48 hr depending on hydration level. Water content was determined from the average difference in weight between the original and dried aliquots. Dried aliquots were further combusted at 510°C for 2.5 hr, and the weight difference between the dry and the LOI-combusted material was the average total organic carbon (TOC) content. Average values and standard deviations for each sample analysis are provided in Table S1.

Sediment grain size was determined in triplicate for each sample after passing air-dried material through sieves for 0.508 mm (phi size, φ, 1.0), 0.381 mm (φ 1.75), 0.2286 mm (φ 2.0), 0.1397 mm (φ 2.75), and 0.1168 mm (φ 3.0). Large shell pieces or organic debris were removed from the largest sieve, and sediment finer than φ 3.0 (e.g., clay) was combined with the smallest size class. All weight measurements were done on a calibrated Denver Instruments balance to ±0.0001 g at least three times. Statistical calculations to describe grain size distribution, and skewness and sorting based on the logarithmic method of moment were done according to Folk and Ward methods using GRADISTAT [49].

### DNA extraction, PCR amplification, and 454 tag pyrosequencing

Total environmental nucleic acids were extracted from all sediment samples based on previously published methods [14], [50]–[52]. Briefly, ∼5 g of sediment were placed in freshly made sucrose lysis buffer (SLB) with lysozyme (1 mg/ml). Samples were harshly vortexed for up to 5 min before incubating at 37°C for 1 hr in a waterbath. After cooling to room temperature and gently mixing with a solution of 5X proteinase K/CTAB/SDS (Fisher Scientific), sediment slurries were incubated overnight at 55°C while shaking at 100 rpm. The solution was allowed to settle, cool to room temperature, and then was transferred to clean tubes and mixed with 10 M ammonium acetate for protein precipitation. After centrifugation, the supernatant was transferred to a set of at least three clean tubes containing 100% cold, molecular grade isopropanol. Because of low biomass overall [estimated from TOC content and based on 2], nucleic acid precipitation was done overnight at −20°C. Nucleic acids were pelleted by centrifugation and washed with ethanol prior to resuspension in Tris-EDTA (TE) buffer. Extractions were stored at −20°C.

DNA extracts were verified by spectrophotometry (Thermo NanoDrop ND1000) and from TBE agarose gel electrophoresis with ethidium bromide staining. Aliquots of triplicate DNA extractions for each of the samples were homogenized and purified for pyrosequencing of the V1–V3 hypervariable region of the 16S rRNA genes, with the forward primer 28F (5′-TTTGATCNTGGCTCAG-3′) and reverse primer 519r (5′-GTNTTACNGCGGCKGCTG-3′), using GS FLX Titanium technology (Roche 454 Life Sciences, Branford, CT, USA) and according to previously described purification and amplification methods at the Research and Testing Laboratory, in Lubbock, TX (USA) [53].

All raw pyrosequences from 112 experimental sample runs obtained from this study were submitted under the NCBI Sequence Read Archive (SRA) Study SRP014466 (http://www.ncbi.nlm.nih.gov). Summaries of the pyrosequencing data, including SRA Accession Numbers for each sample, are included in Table S2.

### Pyrosequence processing, clustering, and taxonomic assignments

Following sequencing, all failed reads, low quality score sequences (<20), and non-bacterial rRNA sequences were removed using the Ribosomal Database Project (RDP) pipeline, version 10 (http://rdp.cme.msu.edu/) [54]. Remaining pyrosequences were trimmed of barcodes and adapters to a minimum length of 170 bp prior to alignment with RDP’s Infernal Secondary Structure Aware Aligner [55]. Following alignment in RDP, chimeric reads were identified using uchime in mothur (www.mothur.org/wiki/Main_Page) (version 1.21.0), and potential chimeric sequences were removed from the dataset after visual examination. Additional pyrosequence screening using mothur included removing sequences with homopolymeric regions and ambiguous reads, and to generate uniform start positions.

Two complementary approaches were used to obtain pyrosequence taxonomic classifications and to identify changes in the dominant members of the communities from the beaches to the genus-level. Pyrosequences were clustered into operational taxonomic units (OTUs) as the basic unit of diversity for general comparisons [56], but major changes in community composition were evaluated after assigning taxonomy [57]. This was done because OTU assignments can be challenging to interpret due to differences in clustering algorithms, potential pyrosequence noise and error, undetected chimera, and our ability to circumvent these problems or remove data without compromising the analyses [57]–[59]. A Phylip distance matrix was created from the RDP Pipeline, which was then used by mothur to cluster pyosequences using the furthest neighbor (i.e., complete-linkage clustering) algorithm that considers all of the sequences in an OTU have a set cut-off distance from all the other sequences in that OTU. This clustering algorithm is also used within the RDP pipeline [54], but it has the potential to overestimate the number of OTUs produced [58], [60], particularly when assigned to specific taxonomic groups [57]. Therefore, OTUs were generated using a range of 3%, 4%, and 5% cut-off criteria for adding pyrosequences to a cluster [58], corresponding to 97%, 96%, and 95% sequencing similarity values, respectively. Non-parametric estimators of OTU richness and diversity were calculated using mothur, including Chao1, Shannon Diversity (H’), and Simpson’s Dominance indices [61]–[63], for the different OTU clustering cut-offs. These values are included in Table S2. A mean value of each index for each sample site was used for comparison. An optimized OTU cut-off of 4% was applied after comparing rarefaction curves (Figure S4) to the number of OTUs, as well as to the Chao1 estimates [64] (Table S2). At this cut-off, rarefaction curves for most (>70%) of the samples approached saturated coverage and a ratio of the calculated OTU number to Chao1 value was >0.6 for approx.70% of the 112 samples. Although it is possible that OTU richness was overestimated, Chao1 values also can underestimate richness [61]. Consequently, the combined approach provided confidence in the relative diversity for the dataset. Taxonomic classifications were done both by taking the filtered reads pre-clustering and uploading them directly to RDP Classifier and also by using mothur and the OTU cluster data and a reference database of known 16S rRNA genes, according to an 80% confidence level [63]. After comparing the results, pyrosequences with specific taxonomic assignments were binned from phylum to genus levels, where possible. The normalized relative abundances (i.e., presence/absence) of each taxonomic unit were used for statistical evaluation of potential diversity changes over time.

### Comparative statistical analysis of community structure and diversity

A distance matrix was constructed using the Bray-Curtis similarity calculation between pairs of normalized presence/absence diversity data with PAST (version 2.14) [65]. The Student’s t-test was done to compare communities from different areas of the beach over time from the Bray-Curtis similarity distances. The unweighted pair group method with arithmetic mean (UPGMA) clustering method (i.e., average-linkage clustering) was also done to examine community similarity of a particular place in time relative to other samples [66]. Bootstrapping with 1000 resamplings provided insight into the robustness of the clustering and cophenetic correlations were determined. To identify the microbial groups that contributed the observed dissimilarity (or similarity) among clusters of data from different locations along the beach transects (e.g., dune versus open beach) or from different sampling times (e.g., May 2010 versus May 2011), similarity percentage (SIMPER) analyses were done [66]. Changes in community composition that could be linked to potential environmental variables were evaluated using iterative nonmetric multidimensional scaling (NMDS) plot analyses constructed from the normalized abundance data and dimensions of geochemical and physical gradients comprising sample ecology [67]–[70]. Environmental dimensions (e.g., depth, pH, grain size, TOC content, and water content) were *log*_10_(*x*+1) transformed to correct potential skewness in the datasets [69]. No correction was done to the index value assigned to the extent of remediation for each sampling time and beach location. Ordination stress of <0.2 was used, with lower stress values representing better matches between sample dissimilarities and dimension ordinations. Non-parametric multivariate analysis of variation (NPMANOVA) calculations after 99,999 permutations were performed to test for significant differences in relative abundances of taxonomic groups from different areas of the beaches or based on environmental variables from the beach transects over time [71]. The non-parametric Spearman’s rho correlation coefficient was used to evaluate correlations among environmental variables and taxonomic groups. Correlations between Shannon diversity (H’) and environmental factors, such as pH, grain size, and water content, were done from coefficient of determination (R^2^) values. Significance was set to ≤0.05, but values from 0.05 to 0.1 were also recorded to indicate weaker significant relationships.

## Results

### Physical changes to the sediments

**Figure 1:**
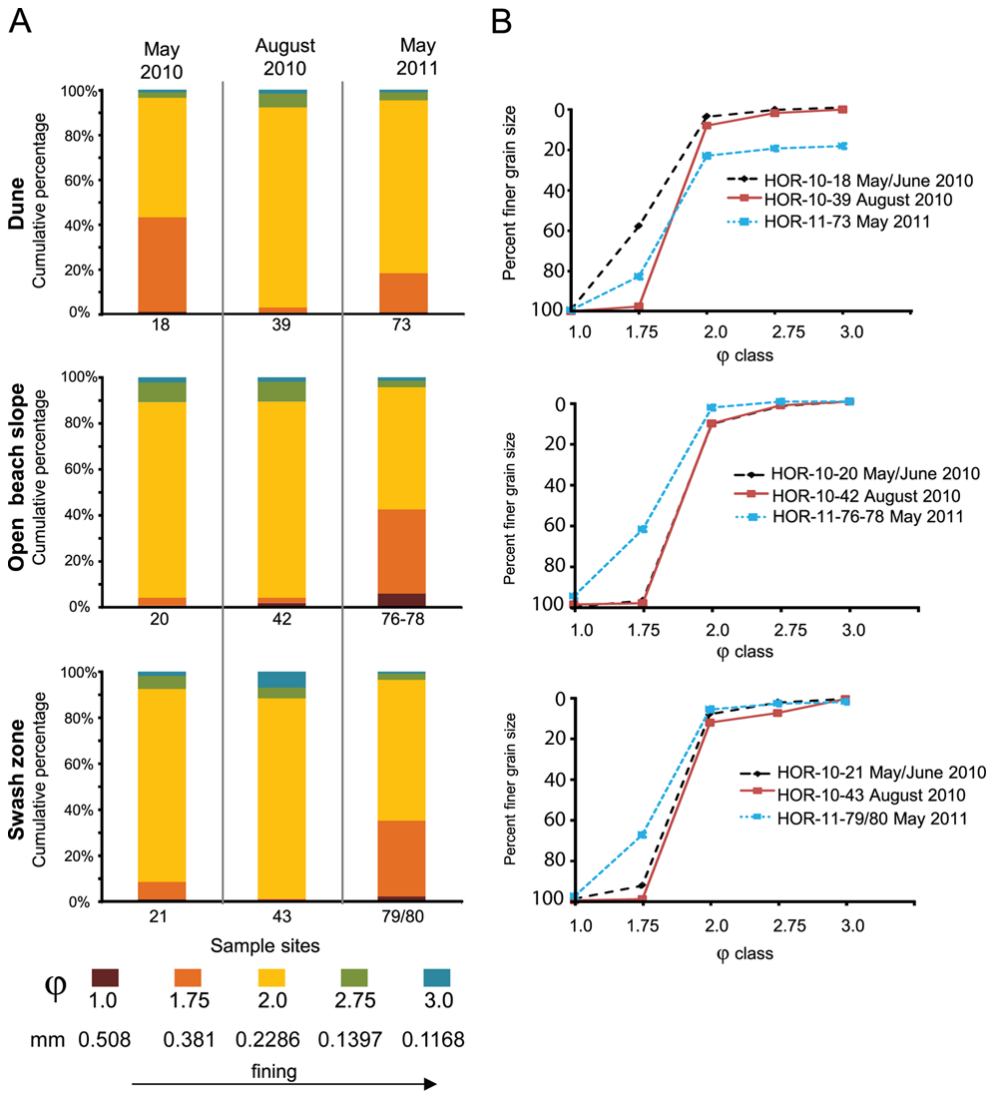
Histograms of grain size distributions for representative sampling sites at Grand Isle. (A) Sites include dune, open beach, and swash zone locations, separated by sampling times. Results from all sample depths for each sample were averaged. (B) Average grain size cumulative frequency distributions for each sample, shown as percent of finer grains.

A transect of approx. 80–100 m from dune to swash zone at Grand Isle was sampled three times, starting May 2010 before moderate levels of oiling and tar ball coverage occurred on the beach (Figure S2). Tar balls and 1–2 cm thick, weathered oil sheets were encountered at the surface through August from the swash and open beach zones of the beach. But, from the sand samples collected over the year, 70.3% had TOC <0.5% (Table S1). Only two (7.4%) of the samples from the surface near ponding water had TOC >1%. Sand was predominately (68.15% ±13.47%) moderately sorted φ 2.0 class size (0.2286 mm) and finely skewed in May 2010. But, between May 2010 and May 2011, sediment became significantly coarser and more poorly sorted on the open beach and swash zone (Figure 1), according to NPMANOVA analysis (F = 14.94, *p*-value = 0.005). Beach-wide sand removal and sand washing occurred on the open beach from late July 2010 until February 2011. Sand was either piled in place where washed, or was removed and new sand was bought to the beach after being washed. Sediment was also tilled and sand from depth was mixed with sand from the surface [19]. Sampling over the winter was not possible because no surface sediment was on the beach at the transect location. From the open beach, variations in sediment water content (F = 2.56, *p*-value = 0.01) and TOC content (F = 4.64, *p*-value = 0.02) were significantly different over time. Variations between the φ 2.0 and φ 1.75 (0.0381 mm) grain sizes were strongly and negatively correlated (Spearman’s rho −0.94, *p*-value = <0.00001), and average TOC content correlated positively to the coarser grain size (rho +0.67, *p*-value = 0.01) and negatively to the finer grain size (rho −0.71, *p*-value = 0.006). The extent of remediation weakly and negatively correlated to the distribution of the finer grain size (φ 2.0) (rho −0.49, *p*-value = 0.08). No other comparisons were significantly different across the beach or between sampling times.

**Figure 2:**
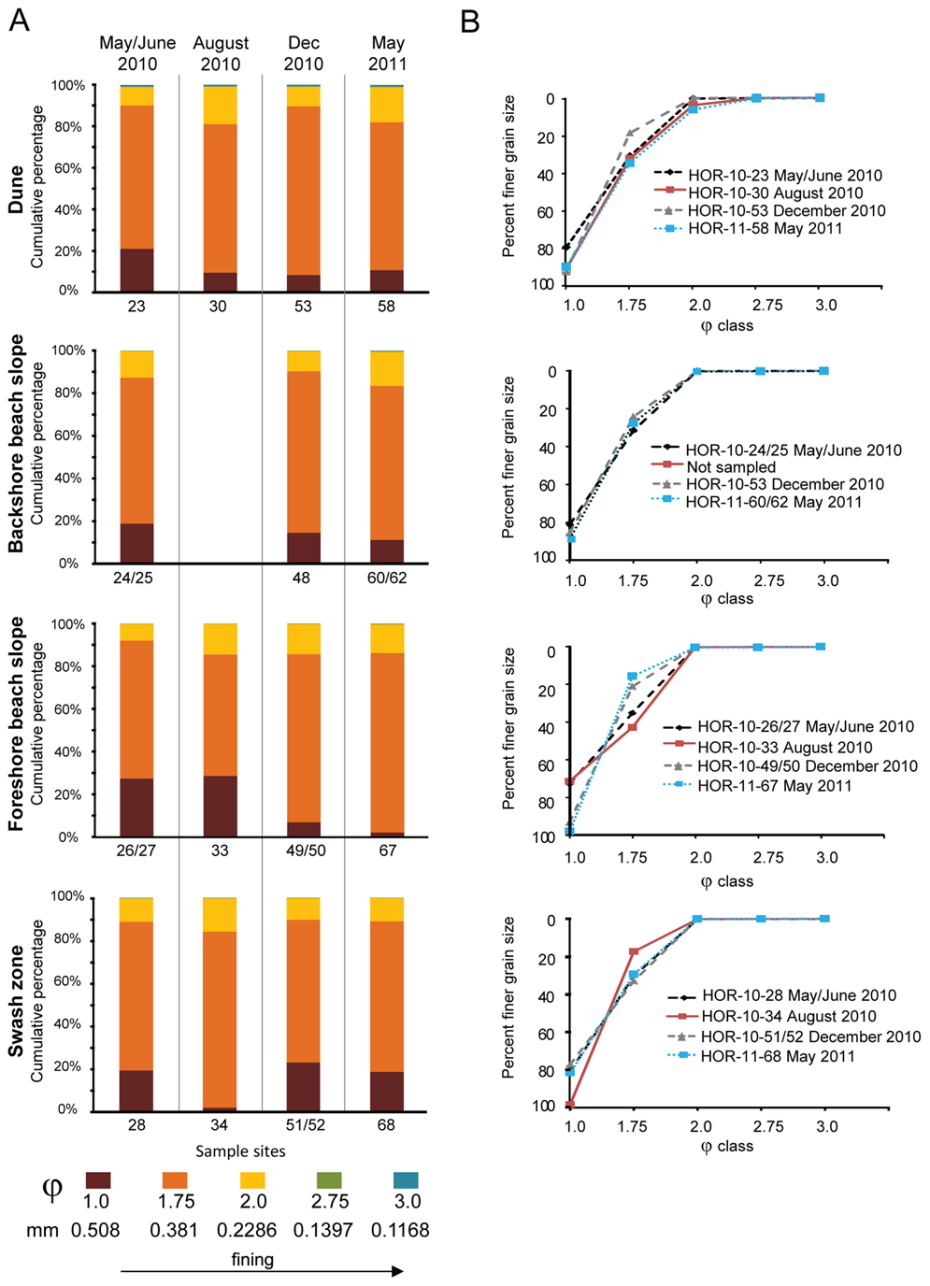
Histograms of grain size distributions for representative sampling sites at Dauphin Island. (A) Sites include dune, backshore beach area, foreshore beach area, and swash zone locations, separated by sampling times. Results from all sample depths for each sample were averaged. (B) Average grain size cumulative frequency distributions for each sample, shown as percent of finer grains.

The beach at Dauphin Island was sampled five times, including twice before tar balls reached the shorelines in May (Figure S3). A transect of approx. 140–200 m from dune to swash zone was sampled. Tar balls were encountered in heterogeneous patches within the swash zone at the sediment surface and floating in the water. Tar balls were rarely found landward of the beach berm at the surface, although tar balls and oily smears were found at depths up to 20 cm over the year in the foreshore areas. Widespread incorporation of oil into the sand was low, as evidenced from 90.6% of all the sand samples at Dauphin Island having <1% TOC concentrations; 60.4% of the samples had <0.1% TOC (Table S1). Only two samples, one from the dune area associated with roots and the other from the swash zone, had TOC content >1%. Sediment was dominated (64.63% ±8.27%) by moderately-sorted, medium-grained to course-grained sand represented by φ 1.75 (0.381 mm) class (Figure 2), which is comparable to the size range reported in historical studies [34]. Dune sand was very well sorted, had a symmetrical distribution, and there were no significant differences in grain sizes over the year. For the backshore sands, significantly different size distributions were noted between June 2010 and August 2010 (F = 3.20, *p*-value* = *0.0006), and between the foreshore June 2010 and all other sampling times. By December 2010, the foreshore sand became more poorly sorted and the grain size distribution went from coarsely to finely skewed based on the addition of finer particles (Figure 2). December 2010 samples were collected after beach-wide sand washing, tilling sand across the beach, and the removal and replacement of sand from the open beach. This physical remediation was most intense at the foreshore. From the swash zone, grain size distributions only varied significantly between the August 2010 and other sampling times (F = 6.46, *p*-value = 0.002), although sorting and skewness did not significantly vary over the year. The distribution of the φ 2.0 class grain size positively correlated to the distribution of φ 1.75 class (rho +0.62, *p*-value = 0.002) but negatively to the coarsest grain size (φ 1.0) (rho −0.43, *p*-value = 0.04). Changes in sediment size influenced sediment water content, TOC content, and pore water pH over time, due to changes in sediment porosity and permeability (Table S1). Water content noticeably increased from the dunes across the open beach to the swash zone, as well as with sampling depth. The coarsest grain size significantly correlated with water content (rho +0.47, *p*-value = 0.02) and negatively to pore water pH (rho −0.43, *p*-value = 0.004). The extent of physical remediation weakly correlated to changes in TOC (rho +0.35, *p*-value = 0.07). According to NPMANOVA tests of data clustered by sampling time, there were only a few significant differences, such as of TOC content between June and December 2010 backshore samples (F = 140.2, *p*-value = 0.006), sediment pH for the dune (F = 3.31, *p*-value = 0.01 between May 2010 and May 2011), the foreshore (F = 4.63, *p*-value = 0.02 between June and December 2010), and swash zone between May 2011 and earlier sampling times (F = 22.19, *p*-value* = *0.05 for June 2010 and *p*-value = 0.02 for August 2010).

**Figure 3:**
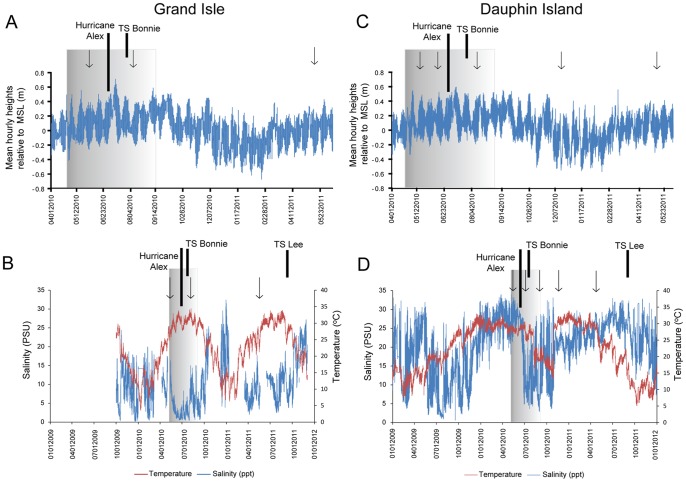
Oceanographic data for Grand Isle and Dauphin Island. Mean hourly tide heights are reported according to mean sea level (MSL) from NOAA’s National Ocean Service station for (A) Grande Isle and (C) Dauphin Island. (B) Ocean salinity and temperature data from the USGS National Water Information System station #07380251 near Grand Isle, and (D) from the Dauphin Island Sea lab, Coastal Marine Station DPHA1. Shaded gray boxes represent the period of time the Macondo Canyon 252 well leaked. Arrows indicate sampling times at each beach. Long bars and shorter bars indicate hurricane or tropical storms (TS), respectively, during the study.

In general, natural processes, such as animal burrowing, cross-shore and long-shore waves, and wind and water transport, cause grain size distribution changes on open beaches. Being predominately influenced by wind-wave energy, mean grain sizes along a beach profile should transition from coarser grain sizes on steeper slopes to finer grain sizes on flatter slopes. Also, open beach sand proximal to the ocean can be reworked from high-frequency and short-duration events, such as high tides from storms [72], [73]. Therefore, differences in sediment grain size and physicochemical properties for specific beach zones at both Grand Isle and Dauphin Island were evaluated in the context of oceanographic and climatic events (Figure 3). Based on the available historical data, conditions at both islands were comparable at the time of the oil spill to previous years, as well as before and after sample collection times. There were also no higher-than-normal average tidal conditions over the year, or extreme excursions in tide heights that could be attributed to Hurricane Alex, which came onshore at the Texas-Mexico border from 29 June –1 July 2010, or for Tropical Storm Bonnie from 24–26 July 2010. A mean maximum tide height of 0.667 m was measured at Grand Isle (Figure 3A) and 0.597 m at Dauphin Island (Figure 3C), both on 7 July 2010. During this same time period at both beaches in July, off-shore salinity was low due to increased freshwater riverine inputs into the areas (Figure 3B and 3D). The beach slope at Grand Isle from the active swash zone to the backshore area was 2 to 4°, and the average high-tide water height could run up the beach to near the backshore area (approx. 60–70 m from the water line). In contrast, the Dauphin Island beach slope ranged from 7–8° from the active swash zone to the berm, and the average high-tide water height did not rise above the berm (approx. 15–20 m from the water line). Consequently, even with storm-enhanced wave and wind energy conditions superimposed onto high-tide water heights [32], wave energy produced by average tidal activity was generally low during the study year and unable to resuspend or transport sediment extensively, or even to bring oil and tar balls inland, on either beach [35]. In summary, natural processes associated with wind and wave activity could not account for increased coarsening and more poorly sorted sediment [74] at both beaches, and mainly in the backshore zones.

### Changes in bacterial diversity over time

**Figure 4:**
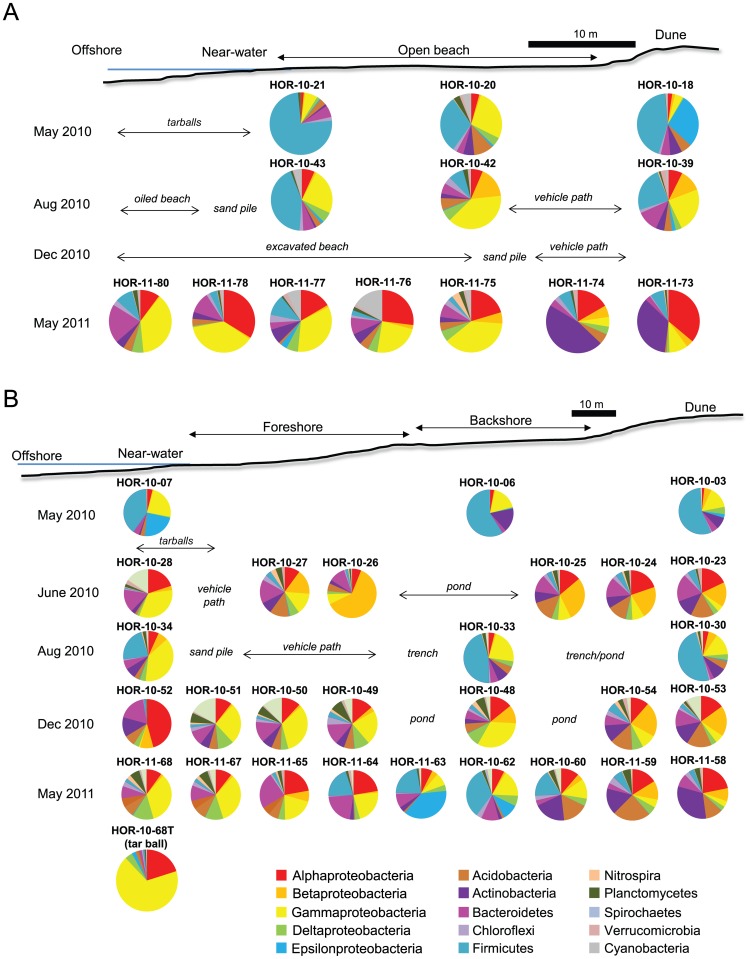
General locations of each sampling site and taxonomic representation along the beach profile. (A) Grand Isle and (B) Dauphin Island. Summaries include only taxa with more than 2% relative abundances (all taxonomic data are included in Tables S3 and S4). Because there were multiple sampling times in May and June at Dauphin Island, composite samples are shown for each month. Remediation activities are schematically included for relevant sampling locations. Photographs from each beach are provided on Figures S2 and S3.

After trimming and screening, 181,710 pyrosequences for three transects at Grand Isle and 373,248 pyrosequences for five transects at Dauphin Island were used for analyses (from 817,175 raw amplicons for both sites) (Table S2). Rarefaction curves showed that OTU richness varied depending on sampling locations at both beaches, with dune samples having saturated coverage and lower numbers of OTUs, open beach samples having more OTUs, and shoreline samples having slightly undersaturated coverage yet generally higher numbers of OTUs (Figure S4 and Table S2). Each of the beach samples were analyzed separately to assess how alpha-diversity varied over time. Although previous studies indicate that microbial diversity in subtidal sandy sediment can change with depth [5], our analyses found that differences in taxonomic diversity with depth were not statistically significant. Therefore, presence/absence data for all depths at each sample location were combined to generate a more representative community composition, and then the data were grouped into sections of the beach according to dune, open beach, and shoreline swash locations for Grand Isle (Figure 4A) and as dune, backshore, foreshore, and shoreline or swash zone locations at Dauphin Island (Figure 4B).

#### Grand Isle

UPGMA cluster analyses of the major taxonomic groups at the class- and phylum-level were done using datasets from the Grand Isle beach transects that were unconstrained (Figure S5A) or constrained (Figure S5B) to sample location. A higher cophenetic correlation coefficient (i.e., distance calculated to be closer to 1) was calculated for the hierarchical cluster tree with samples unconstrained to location (0.88, compared to 0.49 for constrained analysis), meaning that samples collected proximal to each other at the same time had more similar compositions. However, bootstrap values supporting some nodes were stronger for constrained analyses because samples collected from comparable beach locations but at different times (e.g., from the open beach) also clustered together. SIMPER indicated that Actinobacteria, Alphaproteobacteria, Gammaproteobacteria, and Firmicutes explained 72% of the cumulative differences between the dune and open beach communities. SIMPER indicated Gammaproteobacteria, Alphaproteobacteria, Firmicutes, and Bacteroidetes explained 76% of the cumulative differences between the open beach and swash zone communities.

**Figure 5:**
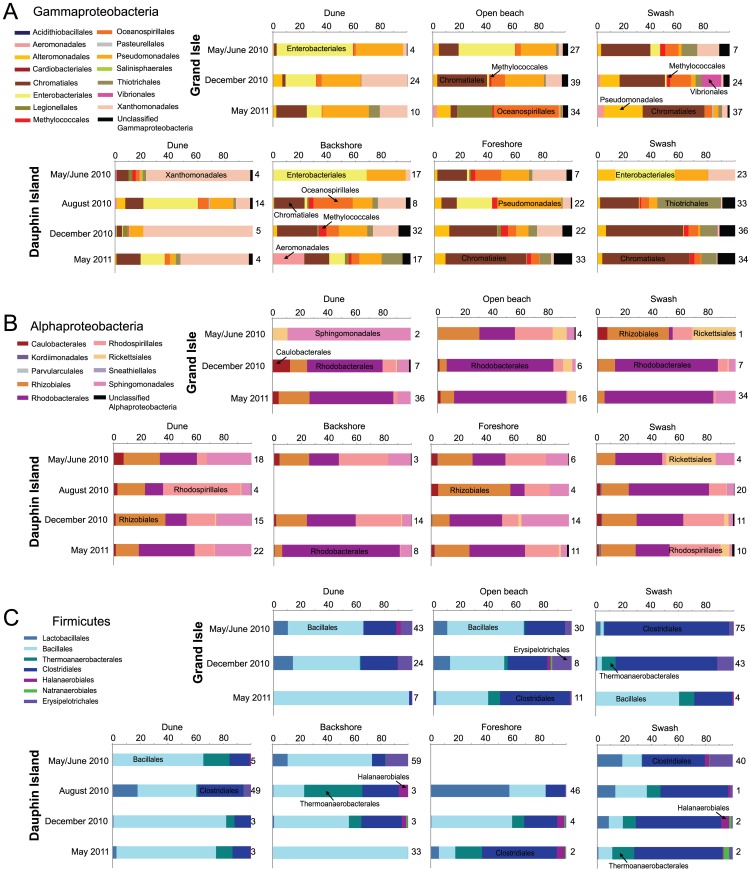
Order-level taxonomic results for Grand Isle and Dauphin Island. Summaries are organized as (A) Gammaproteobacteria, (B) Alphaproteobacteria, and (C) Firmicutes**,** by beach, sample location, and sampling time. Numbers included to right of each bar represent the percentages that the class or phylum represented in the complete dataset for that sample, at that specific sampling time and location.

The open beach and swash zone sediments were dominated by Gammaproteobacteria (Table S3). The gammaproteobacterial relative abundances from the dunes and open beach were statistically significant (NPMANOVA F = 5.92, *p*-value = 0.02), with distinct changes at the order-level (Figure 5A) and genus-level (Figure S7A) for all areas of the beach over time. The May 2010 open beach was dominated by Enterobacteriales, particularly *Escherichia*/*Shigella* and *Serratia* spp. (Figure S7A). Dune samples in May 2010 through May 2011 also had high relative abundances of Enterobacteriales, but abundances decreased over time while Xanthomonadales and Chromatiales abundances increased. The proportion of Pseudomonadales remained constant at the dune but decreased in the open beach samples. Also from May 2010 to December 2010, the relative abundances of Enterobacteriales decreased at the open beach, but Legionellales (*Legionella* spp.) increased. There was a marked increase in Vibrionales (*Photobacterium* spp.) at the swash zone during December 2010 compared to other sampling times and any other beach location (Figure S7). Oceanospirillales abundances increased significantly over time, with the highest values from the May 2011 open beach, such as among the *Litoricola* and *Alcanivorax* spp. Other groups with increased relative abundances from August 2010 to May 2011 were Alteromonadales, including the genera *Alteromonas* and *Marinobacter* spp., and Chromatiales, such as *Rheinheimera* spp. (Figures S5).

There were also distinct differences among the Alphaproteobacteria from different areas of the beach, including that their overall abundances increased significantly over time for all areas of the beach (Figure 5B). Alphaproteobacterial relative abundances significantly and negatively correlated to the Firmicutes (rho −0.75, *p*-value = 0.003) and Epsilonproteobacteria (rho −0.65, *p*-value = 0.02). At the open beach, relative abundances of Rhodobacterales increased nearly 4X, with the most notable increase being from the *Oceanicola* spp. Moreover, Rhizobiales and Rhodospirillales at the swash zone had decreased relative abundances, while Rhodobacterales and Sphingomonadales had increased abundances. The distribution of Alphaproteobacteria strongly correlated to the extent of remediation at the beach (rho +0.91, *p*-value = <0.00001).

There were notable abundance differences among the Firmicutes, with representation along the beach profile generally decreasing through time (Figure 5C). At the swash zone, Bacillales increased from barely above detection in May and December 2010 to more than 60% of the community by May 2011, although the overall abundances of all Firmicutes decreased from 75% to 4% for the whole community (Table S2). This was a commensurate decrease in Clostridiales for the same time periods, where this group previously represented >70–80% of the communities in May/June and December 2010. The distribution of Firmicutes negatively correlated to the extent of remediation at the beach (rho −0.79, *p*-value = 0.001).

There were also significant differences in the relative abundance of Actinobacteria from the dunes, open beach, and swash zones (NPMANOVA F = 3.56, *p*-value = 0.02). The distribution of Actinobacteria negatively correlated with sediment water content (rho −0.55, *p*-value = 0.05). The distributions of other groups across the beach profile over time were not significant.

#### Dauphin Island

UPGMA cluster analyses of Dauphin Island beach transect data unconstrained (Figure S6A) by location had a higher calculated cophenetic correlation coefficient (0.83) (Figure S6B) compared to data constrained by location (coph. corr. 0.59; Figure S6B), although stronger bootstrap values were produced for location-constrained clustering. Like the Grand Isle UPGMA results, clusters of similar communities formed with proximal locations, regardless of sample times. Other clusters formed with samples from locations not necessarily proximal to each other, such as for the foreshore and swash zone sites. SIMPER analyses indicated that the dune communities differed from other beach locations because of the relative abundances of Actinobacteria.

According to NPMANOVA tests, Actinobacteria relative abundances at the dunes significantly differed between the backshore (F = 4.5, *p*-value = 0.05), foreshore (F = 9.85, *p*-value = 0.006), and the swash zone (F = 20.65, *p*-value = 0.008). The distribution of Actinobacteria significantly and negatively correlated with pore water content (rho −0.65, *p*-value = 0.008), TOC content (rho −0.69, *p*-value = 0.007), and pore water pH (rho −0.70, *p*-value = 0.0002). At the dune, 62% of the overall community variation compared to the backshore could be explained by Actinobacteria combined with Gammaproteobacteria, Firmicutes, Betaproteobacteria, and Acidobacteria.

Relative abundances of Gammaproteobacteria, Firmicutes, Betaproteobacteria, Bacteroidetes, and Epsilonproteobacteria explained 65% of the differences between backshore and foreshore communities, and Gammaproteobacteria, Firmicutes, Betaproteobacteria, Bacteroidetes, and Deltaproteobacteria explained 68% of the differences in the foreshore and swash zone communities. Overall, Gammaproteobacteria represented a significant portion of the foreshore and backshore open beach and swash zone areas (Figure 4B; Table S4), although representation among the gammaproteobacterial groups, depending on location along the beach transects, changed over time (Figure 5A; Figure S7B). The distribution of Gammaproteobacteria positively correlated to pore water content (rho +0.62, *p*-value = 0.001), TOC (rho +0.65, *p*-value = 0.0009), and weakly to the extent of remediation at the beach (rho +0.37, *p*-value = 0.08). The distribution of Gammaproteobacteria negatively correlated to the distribution of Betaproteobacteria (rho −0.63, p-value = 0.001) and Acidobacteria (rho −0.56, p-value = 0.005), and positively to Spirochaetes (rho +0.54, p-value = 0.0008) and Nitrospira (rho +0.42, +p-value = 0.05). From May and June 2010 sampling times, more than 40% of the Gammaproteobacteria were Enterobacteriales and ∼20% were Xanthomonadales. These groups (along with Epsilonproteobacteria) were undetected later in the study from the swash zone (Figure 5A), although representation among the Epsilonproteobacteria spiked in May 2011 open beach samples where there was a back-beach pond (Figure 4B). Incidentally, Epsilonproteobacteria from the Campylobacterales order, dominated by *Helicobacter* spp. that represented >45% of the community, were prevalent within the swash zone in May 2010, further indicating potential fecal contamination of the beach [75]. Thiotrichales became the most abundant gammaproteobacterial group in August 2010, being represented by predominately Piscirickettsiaceae (e.g., *Methylophaga* spp.) and Thiotrichaceae (e.g., *Leucothrix* spp.) (Figure S7B). The relative abundance of Thiotrichales decreased over time, but the relative abundance of *Chromatiales* increased, predominately among the Ectothiorhodospiraceae (e.g., *Ectothiorhodosinus* spp.). *Ectothiorhodosinus* spp. only represented ∼11% of all Gammaproteobacteria in May and June 2010 foreshore samples, but dominated December 2010 and May 2011 foreshore sediments at 19% and 41%, respectively, compared to other gammaproteobacterial groups (Figure 5A and Figure S7B). In the backshore sediments, Ectothiorhodospiraceae were undetected in May/June 2010, but represented ∼11% of the community by August 2010 and became the most abundant gammaproteobacterial group by December 2010 (Figure S7B). Foreshore sediments were dominated by Pseudomonadales in August 2010, but this group decreased to nearly undetectable levels by May 2011.

According to NPMANOVA tests, the relative abundances of Betaproteobacteria between the swash zone and dune (F = 16.42, *p*-value = 0.01) and between the swash zone and backshore (F = 4.66, *p*-value 0.04) were significantly different, although SIMPER indicated that the greatest difference in the community from the swash zone and all other sampling locations, regardless of sampling time, was explained by the relative abundances of Gammaproteobacteria. The distribution of Alphaproteobacteria positively correlated to the distribution of Verrucomicrobia (rho +0.56, *p*-value = 0.005) and Bacteroidetes (rho +0.55, *p*-value = 0.01). Among the Alphaproteobacteria, relative abundances of Rhodospirillales increased in the swash zone, represented predominately by *Pelagibius* spp. within the Rhodospirillaceae (Figure 5B). The December 2010 foreshore and backshore samples had the highest Rhodobacterales abundances, with representation from a variety of genera, including *Paracoccus*, *Albidovulum*, *Loktanella*, and *Rhodobacter* spp. These groups had low relative abundances in May/June 2010.

The distribution of Firmicutes negatively correlated to Nitrospira distributions (rho −0.54, *p*-value = 0.01), and Firmicutes comprised a significant portion of the dune communities from all depths. But, like Grand Isle, representation among Firmicutes dropped from the backshore, foreshore, and swash zones over time (Figure 5C). Variability at the order-level suggested that Bacillales were prevalent from dune and backshore samples, but Clostridiales were more prevalent from foreshore and swash zone areas from earlier sampling times.

### Physicochemical controls on community composition

**Figure 6:**
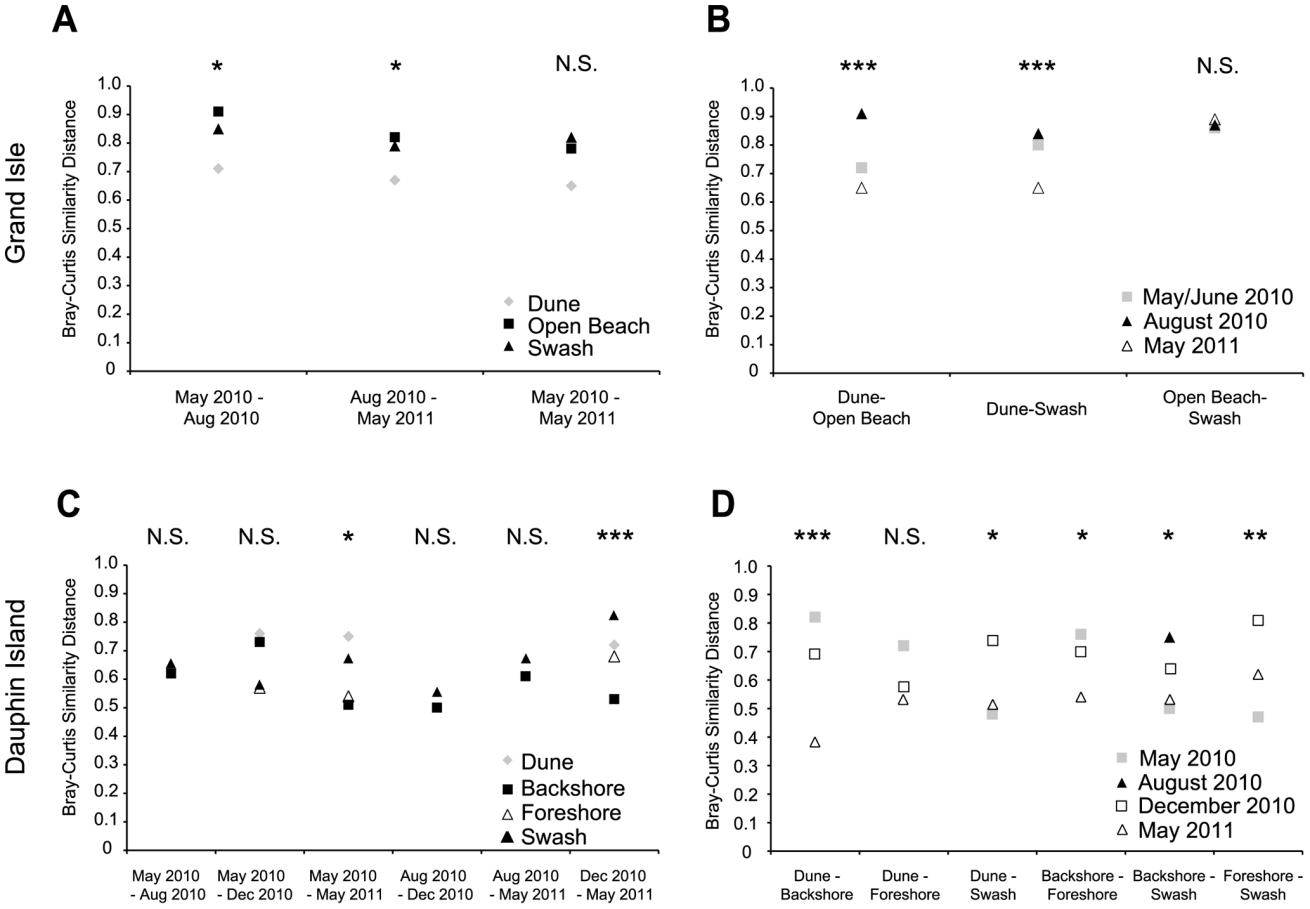
Bray-Curtis similarity distances among groups of samples for sampling times and beach locations. For Grand Isle, comparisons are (A) between different sampling times and (B) between different locations along the beach profile, and for Dauphin Island comparisons are (C) between different sampling times and (D) between different locations along the beach profile. N.S. = comparisons were not significant. *weakly significant, between *p*-values 0.051 to 0.1; **significant, between 0.0001 to 0.05; ***highly significant, <0.0001.

Bray-Curtis similarity distances were used to evaluate community compositional changes across time and from sampling locations for Grand Isle and Dauphin Island (Figure 6). At Grand Isle, the dune community compositions did not vary significantly over time, but the differences between communities from the dune and open beach did, as did the communities from the dune and swash zone (Figure 6A). But, comparison of open beach and swash zone communities were not significantly different, meaning that the two communities were similar to each other over time (Figure 6B). Collectively, these results indicate that most of the community compositional changes were from the open beach and swash zones. At Dauphin Island, the dune community composition did not vary over time (Figure 6C), but similarity distances between the dune communities and the other sampling sites decreased over time (Figure 6D). The dune and backshore communities were initially highly similar in May 2010, which is expected because the sampling locations were proximal to one another. However, with time, the communities diverged and did not recover similar community compositions. The dune and the foreshore samples were less similar to one another initially, which is expected given the physical distance between the sites. Over time, the community compositional differences increased. Dune and swash zone community compositions were initially dissimilar, but by August 2010 the communities were more similar to each other, until May 2011 when similarity returned to May 2010 values. The backshore and foreshore communities became more dissimilar over time, but the swash zone and backshore or foreshore areas became more similar.

**Figure 7:**
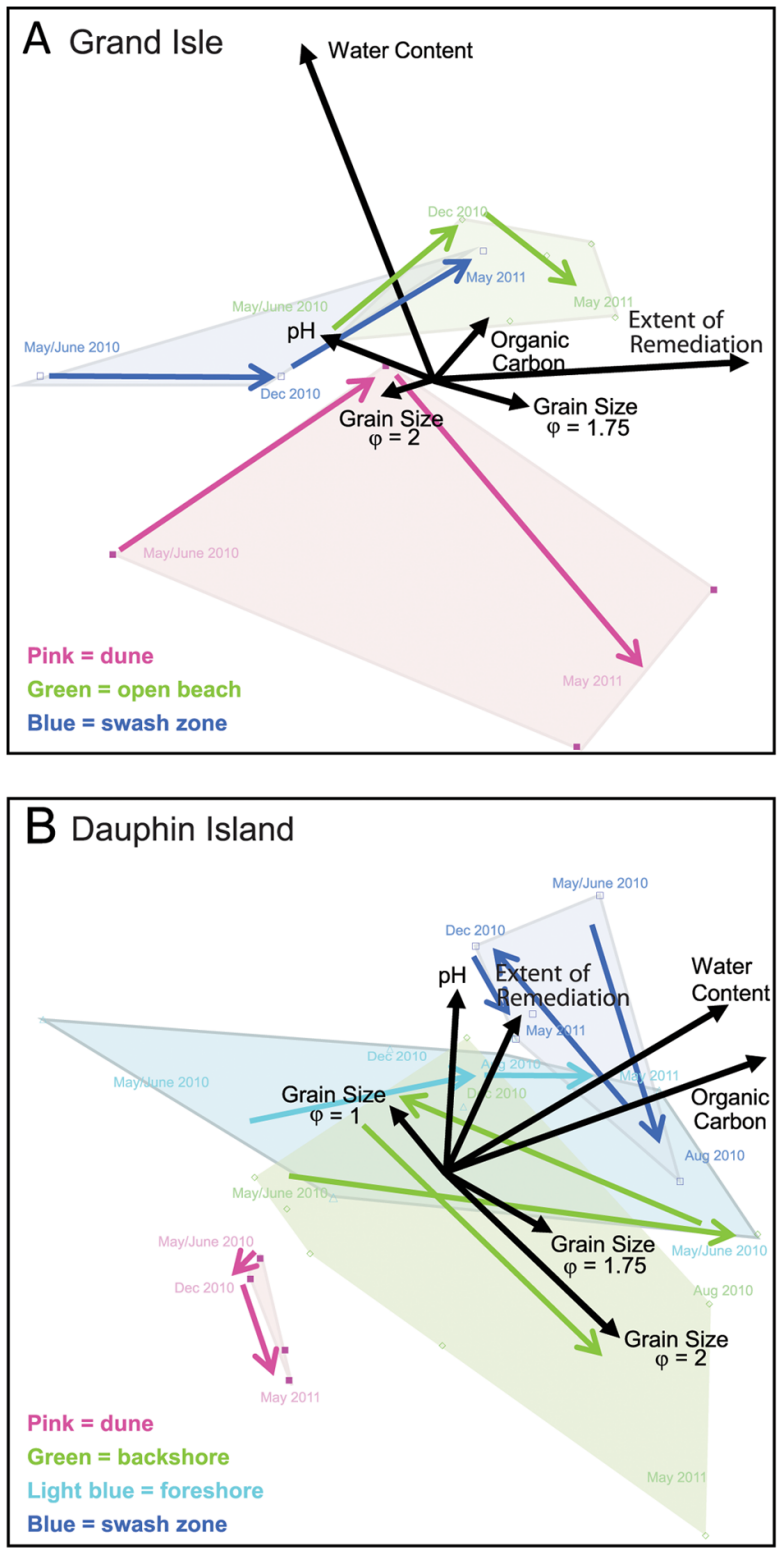
Non-metric multidimensional scaling (NMDS) plots based on microbial community compositions for all beach sand. (A) Grand Isle and (B) Dauphin Island community compositions from 16S rRNA gene pyrosequences that correspond to environmental variables and the extent of physical remediation at the beaches, shown as vectors. For (A), stress = 0.11, axis 1 R^2^ = 0.61 and axis 2 = 0.35. For (B), stress = 0.16, axis 1 R^2^ = 064 and axis 2 = 0.23. Colored arrows indicate the temporal shifts in community ordination for the dune, open beach areas, and swash zone.

To evaluate the potential controls that environmental parameters may have exerted on community composition at particular locations along the beach transects, physicochemical parameters from both beaches were used for NMDS analyses of taxonomic data. From Figure 7, communities closer to one another in NMDS space are similar to each other, and movement across NMDS space in directions (Figure 7, colored arrows) consistent with vectors (Figure 7, black arrows) was interpreted to be influenced by the parameter(s), corresponding to significance from Spearman’s rho values. At Grand Isle, the NMDS plot confirmed that communities from different locations along the beach were distinct from each other initially (Figure 7A). Community ordination of the open beach and swash zone samples shifted across NMDS space from May/June 2010 to December 2010 toward the influence of grain size φ 1.75, the coarser size, as well as ordination of the extent of remediation vector. But, from December 2010 to May 2011, the dune community shifted away from water content, which corresponded to the fact that these samples were drier than earlier sampling times (Table S1). The open beach and swash zone communities shifted in directions of coarsening grain size and organic carbon and/or water content. Shift across NMDS space also corresponded to the vector direction for remediation extent.

Similarly, the NMDS analyses for Dauphin Island (Figure 7B) indicated that dune communities were distinct from swash zone communities, and that some foreshore and backshore communities were similar to each other at all times, which corresponded to the Bray-Curtis similarity distance comparisons. The swash zone community compositions were more similar to the foreshore communities, which also corresponded to the other analyses. Most of the community compositional changes in NMDS space were for backshore and foreshore samples, showing that community ordination shifted in the directions of water and carbon content vectors, and perhaps the extent of remediation. For the backshore communities, the May 2011 samples were affected by changes in grain size, which corresponded to finer grain size. But, the foreshore communities shifted in NMDS space from August 2010 to May 2011 in the direction influenced by higher abundances of coarser size. Changes in backshore community ordination were consistent with directions influenced by grain size more than the other variables.

**Figure 8:**
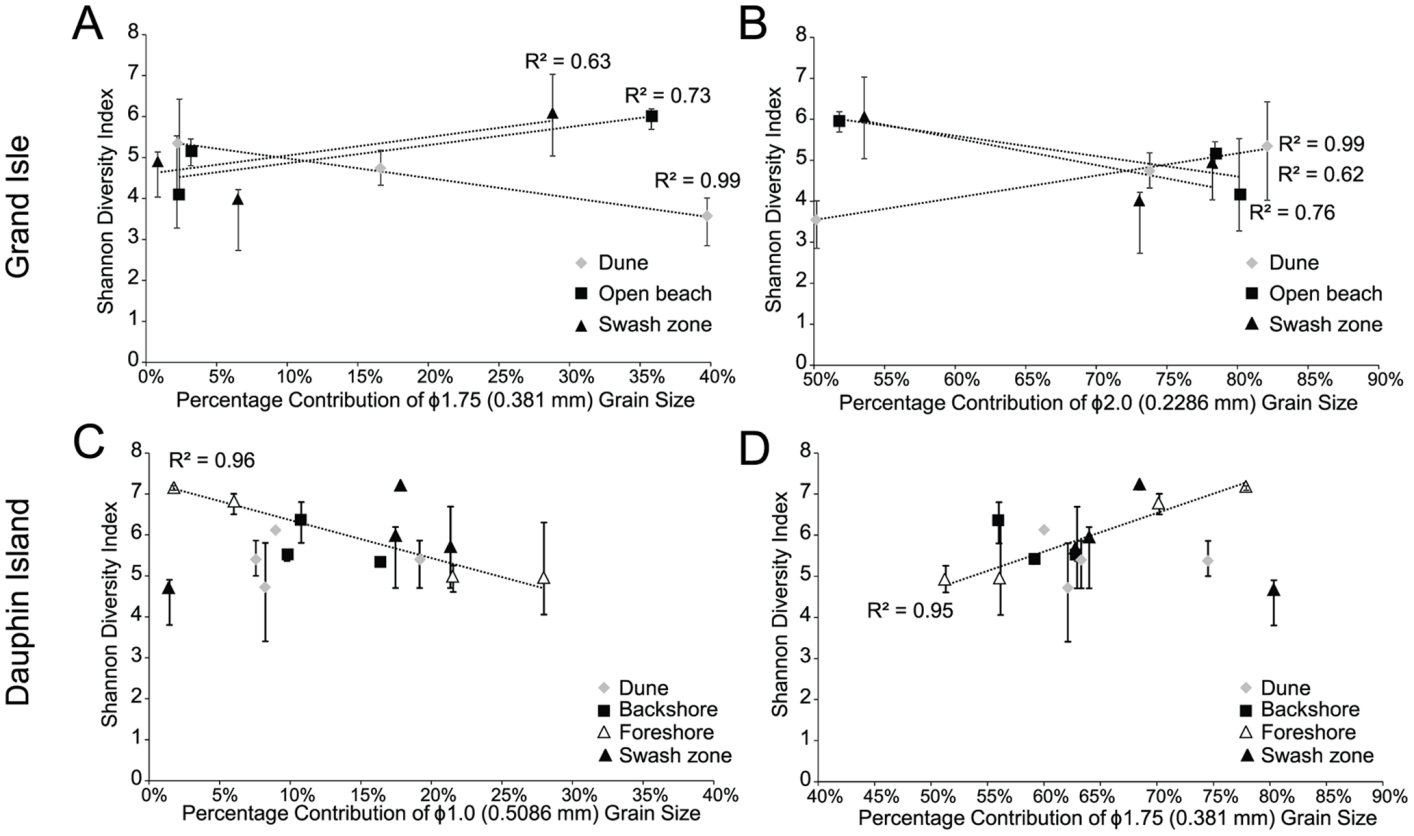
Changes in the percent contribution of different grain sizes compared to Shannon Diversity (H’) index values. Diversity was calculated from OTUs for (A) and (B) Grand Isle, and (C) and (D) Dauphin Island. (A) and (C) compare the coarser grain size contribution, and (B) and (D) compare the finer grain size contribution.

From these results, differences in grain size, as well as extent of remediation efforts, appeared to explain most of the community composition variability for the beaches, particularly for open beach zones. Differences in grain size distribution were compared to H’ indices derived from OTU richness. For Grand Isle (Figure 8A and 8B), higher dune microbial diversity based on H’ values strongly correlated to finer sediment grain sizes (R^2^ = 0.99, *p*-value <0.005), but diversity at open beach and swash zones was higher with increased coarser grain size abundance, although the relationships were not as strong (R^2^∼0.7 and 0.6, respectively, *p*-values >0.05). At Dauphin Island (Figure 8C and 8D), higher H’ values significantly correlated to increased contributions by finer grain sizes only for foreshore samples (R^2^∼0.9, *p*-value = 0.001). Grand Isle dune and open beach microbial diversity based on H’ values decreased with increased TOC content (R^2^∼0.8, *p*-value = 0.007), whereas higher diversity from Dauphin Island dune and backshore samples correlated to increased TOC content. Despite the indication from other statistical analyses that sediment water content and pore water pH may explain changes in microbial diversity at particular areas of the beaches over time, no statistically significant correlations with H’ values resulted.

## Discussion

Microorganisms in coastal habitats like sandy beaches are important to cycling terrestrial and marine materials (e.g., [5], [6], [8], [76]). The potential exists for indigenous microbes in sandy beaches to play critical biogeochemical roles within supratidal sandy beach ecosystems, but there is limited knowledge of microbial community structure and function from many beaches, particularly along the Gulf of Mexico. From descriptions of sandy beach microbial diversity proximal to or within the swash zone from several Gulf coast beaches following the *Deepwater Horizon* oil spill, Newton et al. [15] express that a greater understanding of backshore, supratidal beach microbial communities is needed. Among the reasons, it is unclear if supratidal habitats comprise communities similar to intertidal and subtidal zones. Supratidal microbial communities are situated in generally nutrient-poor areas that do not receive constant or even sporadic input from ocean water. Therefore, it is possible that supratidal microbial communities may be more sensitive to perturbations from extreme changes in water availability (e.g., inundation by marine water during storm events, desiccation) [6] and nutrient and organic carbon content introduction or depletion [7], including from oil contamination. The results from our study of two beaches indicate that microbial community compositions across the supratidal zones are distinct from each, with higher community similarities for areas proximal to each other. Also, there is clear evidence that supratidal and subtidal microbial communities are dissimilar to each other. Together, these findings improve our understanding of supratidal beach ecosystem functional diversity and of the potential to evaluate beach ecosystem resiliency following a disturbance.

### Processes affecting sediment distribution and microbial communities on sandy beaches

A variety of processes influence the distribution, composition, sorting, and overall depositional history of sand on beaches. These processes also affect the environmental heterogeneity of the sand habitat, which have consequences on the structure and distribution of microbial communities within the different niches [5]. From previous work, one can predict that potential associations with vegetation and rhizosphere development, and biostabilization due to microbial mat or biofilm development may influence microbial community stability in some areas of the beach, such as in dunes. Also, swash zone sediment and their microbial communities should be distinct from backshore to foreshore open beach areas based on the proximity to water laterally and at depth. Daily tidal and wind-wave energy events replenish subtidal microbial communities with saline fluids and nutrients, and the wind-wave energy also contributes to sediment deposition and reworking, homogenization, or even the removal of previously deposited material [14], [70], [74], [77], [78]. In contrast, because of reduced interactions with oceanic fluids, open beach supratidal sands should be reworked infrequently by tropical storms, hurricanes, and other events like seasonal cold fronts [74]. Therefore, from limited fluid interactions, supratidal sand habitats can have higher evaporation and desiccation potential at shallow sediment depths, and variable pore-water pH and redox conditions [1].

At the most basic level, sediment grain size distributions influence reactive surface area, surface area for colonization, and pore and gas-fluid diffusion and exchange because of differences in sediment porosity and permeability, nutrient and carbon availability, pore water pH; grain size also affect the ability for wind- or water-driven transport and potential protection against predation [79], [80]. In terrestrial soils, to which supratidal beach settings may be analogous, grain size can have a greater impact on microbial diversity and community structure than other factors, like pH and the type or amount of organic matter input [80]. Many previous studies demonstrate that microbial diversity of terrestrial soils [7], [79], [80] and marine sediments [11], [73], [81] significantly correlates to sediment size. From our analyses, we could expect that changes in microbial community composition over time would significantly correlate to changes in grain size distribution on the beach.

However, mean grain size changed along the beach profiles that did not correspond to known factors that should affect grain size distribution. Typically, there is a transition from coarser gain sizes on steeper beach slopes to finer grain sizes on flatter slopes, and grain size distribution also do not change in backshore open beach areas generally above the maximum high tide or storm level heights [72], [73]. Meteorological and oceanographic data for both beaches suggest that tidal ranges and wind-driven wave energy patterns due to storm events, or even above-average tidal activity, could not explain why sediment from supratidal areas became more poorly sorted and had variable sand size distributions over the year (Figures 1 and 2). In the absence of natural processes that could explain changes to the sediment packages [74], we suggest that the oil spill emergency response, including physical remediation on the public beaches, altered sand grain size distributions and sorting along the open beaches. Grain size changes strongly correlated to the extent of remediation on a beach over time.

Consequently, grain size changes induced microbial community compositional shifts (Figures 4, 6, and 7). Beach renovation following the import of new sand can potentially improve beach sand and water quality, particularly due to the removal or dilution of pathogenic groups. But, beach renovation can also diminish an ecosystem’s beneficial microbes and alter ecosystem function [82]. If sand was completely removed from a beach and replaced with material from a different area of that beach, or from a completely dissimilar beach system, then this replacement or replenishment process could cause changes to sediment grain size distribution, as well as result in changes to the microbial community compositions for a particular area of the beach. Instead of complete removal of sand, beach remediation can be done by tilling, homogenization, and sand washing. Sand washing involves the use of ocean water, sometimes heated to high temperatures, and has the potential to separate finer grain sizes from a sediment package due to winnowing [19]. Hypothetically, utilization of ocean water could replace microbial biomass from sediment pore spaces or adhered to grain surfaces. A different suite of microbes, perhaps with higher affinity to marine planktonic groups, may colonize the washed sediment after the sand is returned to a beach. Sand washing might also cause microbial community compositions to become more similar to each other, particularly for areas of a beach that were previously dissimilar, such as from the backshore and swash zones, over relatively short periods of time.

According to Gundlach and Hayes [83], whole-scale removal of oil contamination from coastal habitats should be done after all potential contamination has beached. These oil spill response recommendations have been assessed with environmental sensitivity or vulnerability indices developed in the late 1970s and 1980s [83] and adopted by numerous agencies recently (e.g., [84]). Other recommendations include that minimal sand should be removed from the beach during cleanup, that mechanical methods to remediate the beaches should be minimal, and that beaches should not be driven over because this could grind unrecovered oil contamination deeper into the sediment and cause compaction. For both Grand Isle and Dauphin Island, *impromptu* roads were made for heavy machinery and small vehicles (e.g., ATVs) in foreshore and backshore open beach areas within days to weeks of beaching oil. Moreover, sand was removed, tilled, and/or washed in place using ocean water within weeks to months after oil came ashore [19], all while more oil was washing onto the beaches. We suggest that these physical remediation activities constitute disturbance to the indigenous supratidal sand microbial communities.

### Assessment of microbial community regime shifts and resiliency

The number of microbiological studies done from coastal and shoreline settings since the *Deepwater Horizon* oil spill [6], [15], [85]–[95] indicate that there is intense interest in understanding microbial diversity and ecology of oil spills. However, of these studies, there have been very few to evaluate supratidal beaches since the *Deepwater Horizon* oil spill [6], [15], [87]. One study done by Lisle and Stellick [87] includes samples collected from shorelines between May 7 and June 16, 2010, including from Grand Isle and Dauphin Island. Changes in microbial diversity patterns were interpreted by using denaturing gradient gel electrophoresis, which meant that no sequence data are available for comparison. Another study by Newton et al. [15] includes four sampling times after the spill in 2010 (June, August, September, November) from seven different Gulf coast beaches, some of which were remediated. Samples originated from the surf zone surface water, intertidal and submerged surface sand within the swash zone, and exposed intertidal surface sand from the beach face. The authors conclude that increased relative abundances of putative hydrocarbon-degrading taxa within the communities over time, for the beach areas sampled, could be attributed to disturbance caused by beach oiling. Although microbial communities were evaluated from pyrosequences, which is methodologically similar to our study, only a small area of the beach could be directly compared for both their and our study. Similar microbial communities were retrieved for the June and August sampling times, suggesting perhaps a common response for the beach microbial communities to potential oiling and remediation efforts. For the backshore open beach and dune areas that we sampled, however, the microbial diversity results are without comparison. Therefore, we used data from the May and June 2010, pre-spill sampling times to provide a baseline to compare all other sampling times for our study and to determine ecosystem resiliency for the full supratidal beach systems.

The pre-spill results indicated that there were few compositional similarities between supratidal and subtidal sand microbial communities, and that each area of the beach had its own distinct community composition (Figure 6). Pre-spill communities were dominated by microbial groups indicative of fecal contamination. Specifically, May and June 2010 Grand Isle swash zone and open beach samples were dominated by Enterobacteriales. Campylobacterales dominated the dune samples. Enterobacteriales and Campylobacterales were also prevalent from May and June 2010 Dauphin Island swash zone and backshore beach samples. In 2010, testing by LDHH identified that an average of 7.5% of Grand Isle samples exceeded state standards for the *Enterococcus* test [39]. Approximately 4.4% of LDHH samples in 2011 exceeded state standards [37]. But, according to the NRDC records, nearly all of the 2010 public beach closures issued at Grand Isle were due to the *Deepwater Horizon* oil spill, with a very small number of closures in 2011 due to the oil spill. US EPA samples from the public beach at Dauphin Island did not exceed Alabama state standards for enterococci monitoring during the study period, although there were 53 public beach advisory days in 2010 due to the *Deepwater Horizon* oil spill [39]. It is highly unlikely that these high relative abundances of fecal indicator bacteria represent indigenous populations in the supratidal beach zones. Recent research from other sandy beaches indicates that these bacteria tend to be transient occupants of sandy beach systems, and that fecal contamination for any length of time on a beach can severely affect beach water quality and overall beach health [75], [96], [97]. However, most agencies do not sample intertidal or supratidal beach sand, only subtidal sand, even though fecal indicator bacteria, such as *E. coli* and *Enterococcus* spp., can have 38X higher densities in wet sand than in open water [98]. Higher recoveries from wetter rather than drier sand [99] may explain the spike in Campylobacterales (e.g., *Campylobacter jejuni*) from the May 2011 backshore at Dauphin Island. This area was formerly associated with a pond in December 2010.

In contrast, microbial communities from both open beaches, including foreshore and backshore areas that were physically remediated, had more similar community compositions after the oil spill than pre-spill. By August 2010, the presence of Enterobacteriales and Campylobacterales decreased from beach sand samples, and representation among diverse gammaproteobacterial groups increased, including among known or putative hydrocarbon-degraders within the Oceanospirillales (e.g., *Alcanivorax*, *Litoricola, Oceanospirillum, and Neptuniibacter* spp.), Xanthomonadales (e.g., *Hydrocarboniphaga*, *Arenimonas* spp.) Pseudomonadales (e.g., *Alkanindiges*, *Pseudomonas* spp.), and Thiotrichales (e.g., *Cycloclasticus*, *Methylophaga* spp.), just to name a few (Figure 5A and Figure S7). Oceanospirillales are aerobic to facultatively anaerobic chemoorganotrophs that are halotolerant or halophilic, and are found in open seawater and marine sediments [100]. Some are phylogenetically related to the organisms identified from the open ocean following the *Deepwater Horizon* incident [93], [101]. The relative abundances of Chromatiales also increased over time for both areas of the beaches, including Ectothiorhodospiraceae (several genera) and Chromatiaceae (e.g., *Rheinheimera* spp.). It is unclear what the significance could be for the elevated abundances for these putative anoxygenic phototrophs, as well as halophilic groups typically associated with alkaline, sulfide-containing conditions [102]. But, within the Alphaproteobacteria, relative abundances of other phototrophic and chemoorganotrophic members of the Rhodobacterales and Rhodospirillales also increased for these open beach locations (Figure 5B); some include known oil degraders [6]. By May 2011, relative abundances of these groups were still elevated compared to the previous year prior to the oil spill.

In general, beaches are not routinely physically remediated if fecal indicator counts exceed state standards [26]. This stands in stark contrast to oil spill emergency response, whereby shoreline recovery of recreational and public beaches typically requires physical remediation, regardless of the level of oil contamination [18], [19], [24], [83], [103], [104]. Therefore, we suggest that the physical remediation of the supratidal beaches, specifically sand washing with ocean water, as well as sand redistribution from tilling, induced regime shifts in the sands from pre-spill fecal indicator microbial communities to the open-ocean marine and putative hydrocarbon-degrading communities after the oil spill and remediation efforts.

An ecological regime shift is initiated from extreme perturbations that abruptly change, and rapidly reconfigure, an ecosystem across multiple trophic levels [22], [29], [105], [106]. Evidence for a regime shift from our study stems from the statistical comparisons of microbial communities retrieved from across the remediated beaches and changes in environmental parameters over time. Microbial community compositions after the oil spill included higher relative abundances of putative hydrocarbon-degrading microbial groups in backshore areas of the beaches. It is difficult to explain the existence of these communities in the backshore, where natural wind-wave processes would not be expected to transport oil or marine fluids with hydrocarbon-degrading microbial populations from the ocean, without considering the impact of remediation on the sediment packages. Moreover, we did not observe smooth changes in community compositions across the analyzed environmental gradients from the beaches (Figure 7). Smooth transitions from one community to or from another, even due to hysteresis effects [105], would be expected if communities transformed from alternative stable states as a consequence of changing conditions, like water content or changes to population densities following an influx of nutrients [29]. Similarly, there does not appear to have been any resistance to change from the pre-spill microbial communities following the initiation of intense physical remediation as part of the oil spill emergency response (Figure 5), which may have been expressed by lingering presences of *E. coli*, *Enterobacter* spp., and other bacteria at low relative abundances through time. These bacterial groups were undetected in physically remediated portions of the beaches (Figure S7), just as other groups were undetected before remediation efforts, such as hydrocarbon-degraders belonging to the Oceanospirillales. In essence, the remediation efforts, from sand washing to homogenizing the sand across the beach by tilling and raking, disturbed the open beach so abruptly, especially the state variables of grain size, TOC, and water content, that there was no time from an ecological perspective to establish alternative stable microbial communities. Lastly, it is imperative to continue to assess our expectations of ecosystem recovery and resiliency [22], [23] following the oil spill because, clearly, returning beach microbial communities to pre-spill compositions comprised of fecal indicator bacteria is undesirable [98]. But, it is unclear from our limited understanding of supratidal sandy beach microbiology what constitutes an indigenous community. Future research should address how physical remediation efforts on beaches, including in the wake of oil spills, affect not only the taxonomic, but also the functional, diversity of microbes in beach ecosystems.

## Supporting Information

Figure S1 **Location map and transect positions for sampling at Grand Isle and Dauphin Island.** Shaded base map from Esri (DeLorme) with locations noted for Grand Isle, Louisiana, and Dauphin Island, Alabama. Inset map, southern United States showing the general locations for each beach within the boxed area.
(PDF)

Figure S2 **Photographs from specific sampling locations at Grand Isle, Louisiana, with schematic of beach profile and remediation activities shown over time.** Generalized remediation activities are also shown on Figure 4. From May 2010, (A) sampling location in the foreshore and swash zone, and (B) looking west along shoreline, with dark areas being tar balls and oceanic debris. From August 2010, (C) tar ball mat in foreshore area of the beach where there was also oil onshore, and (D) looking east along the beach of raked and tilled sand, and piles of sand. There was no sample collection December 2010 because the beach was excavated and there were extensive piles of sand on the open beach. From May 2011, (E) sampling the foreshore, (F) looking west along the beach on open beach, and (G) looking west at the backshore beach where there was still a vehicle path along dune face. All photographs were taken by A.S.E.
(TIF)

Figure S3 **Photographs from specific sampling locations at Dauphin Island, Alabama, with schematic of beach profile and remediation activities shown over time.** Generalized remediation activities are also shown on Figure 4. From May and June 2010, (A) looking south from the swash zone, which had tar balls floating in the water and washing up onshore, (B) foreshore area with vehicle path and remediation crew, (C) looking northeast at the backshore of the open beach, showing pond and dunes, and (D) looking south toward the shoreline from the dunes. From August 2010 sampling, (E) large piles of sand were put along the foreshore open beach, behind which was a wide vehicle path, and (F) looking west down the beach from the backshore at deep vehicle tracks. From December 2010 sampling, (G) a vehicle path with deep tracks along the foreshore area comparable to the location of photograph B. From May 2011, (H) looking south from the backshore to the foreshore open beach were there were longer vehicle tracks, and (I) looking north toward the dunes at the open beach. All photographs were taken by A.S.E.
(TIF)

Figure S4 **Rarefaction curves for sediment samples from Grand Isle and Dauphin Island.** (A) – (C), Grand Isle samples, and (D) – (G) for Dauphin Island samples, summarized for different areas of the beach profiles and all depths for each sampling time. The number of OTUs corresponds to 96% sequence similarity clusters. Refer to text for more pyrosequence processing information.
(TIF)

Figure S5 **Dendrograms for unweighted pair-group average (UPGMA) hierarchical clustering, or average linkage clustering for sediment communities from Grand Isle samples.** (A) UPGMA clustering, or average linkage clustering, constrained by sampling time, from June 2010– May 2011, and (B) UPGMA clustering constrained by location from sampling transects. Clustering was done to evaluate if samples from the same places along the beach, and adjacent to each other, would be more similar. For both dendrograms, clustering was based on Bray-Curtis dissimilarities from normalized abundance data for each taxonomic group, and bootstrap values (in %) for 1000 replicates are given at the nodes (>50%). The cophenetic correlation was 0.8787 for (A) and 0.4902 for (B), suggesting that constraining the cluster analysis to sample location does not yield strong similarities because processes acting on bacterial communities at a particular location may be more similar in adjacent locations than to changes occurring within that location at a particular time. Pie charts for each sample location correspond to Figure 4A.
(TIF)

Figure S6 **Dendrograms for unweighted pair-group average (UPGMA) hierarchical clustering, or average linkage clustering for sediment communities Dauphin Island samples.** (A) UPGMA clustering, or average linkage clustering, constrained by sampling time, from June 2010– May 2011, and (B) UPGMA clustering constrained by location from sampling transects. Clustering was done to evaluate if samples from the same places along the beach, and adjacent to each other, would be more similar. For both dendrograms, clustering was based on Bray-Curtis dissimilarities from normalized abundance data for each taxonomic group, and bootstrap values (in %) for 1000 replicates are given at the nodes (>50%). The cophenetic correlation was 0.8338 for (A) and 0.5885 for (B), suggesting that constraining the cluster analysis to sample location does not yield strong similarities because processes acting on bacterial communities at a particular location may be more similar in adjacent locations than to changes occurring within that location at a particular time. Pie charts for each sample location correspond to Figure 4B.
(TIF)

Figure S7 **Genus-level taxonomic results for** ***Gammaproteobacteria*** **.** Summaries are organized by sampling time and by beach location for (A) Grand Isle and (B) Dauphin Island.
(TIF)

Table S1 **Location and physicochemical data for each of the sediment samples collected from Grand Isle, Louisiana, and Dauphin Island, Alabama, 2010–2011.** Grand Isle samples are shaded in gray. N.M. = not measured.
(PDF)

Table S2 **Summary of pyrosequencing data for each of the sediment samples collected from Grand Isle, Louisiana, and Dauphin Island, Alabama, 2010–2011.** Grand Isle samples are shaded in gray. Operational Taxonomic Units (OTUs) and diversity indices were calculated using MOTHUR.
(PDF)

Table S3 **Summary of the number of pyrosequences obtained for each major taxonomic division for sediment samples collected from Grand Isle, Louisiana, 2010–2011.**
(PDF)

Table S4 **Summary of the number of pyrosequences obtained for each major taxonomic division for sediment samples collected from Dauphin Island, Alabama, 2010–2011.**
(PDF)
