# Supplementary figures and images for: Regime Shift in Sandy Beach Microbial Communities following Deepwater Horizon Oil Spill Remediation Efforts
Source: PLoS One. 2014 Jul 18;9(7):e102934. doi: 10.1371/journal.pone.0102934 (PMC4103866; doi:10.1371/journal.pone.0102934)

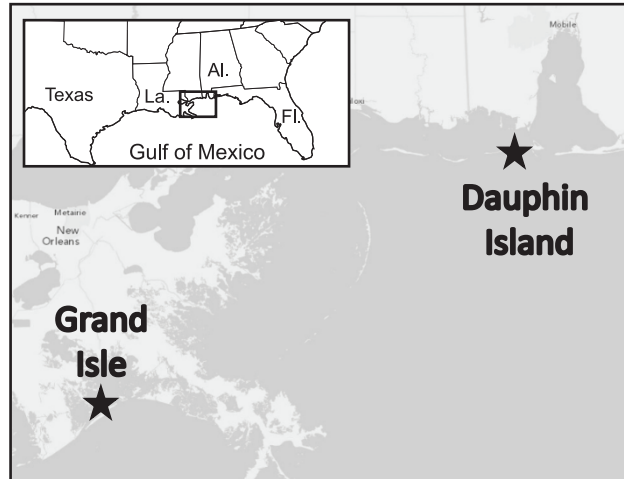

Supplement: Figure S1 — Location map and transect positions for sampling at Grand Isle and Dauphin Island. Shaded base map from Esri (DeLorme) with locations noted for Grand Isle, Louisiana, and Dauphin Island, Alabama. Inset map, southern United States showing the general locations for each beach within the boxed area. (PDF) [file pone.0102934.s001.pdf]

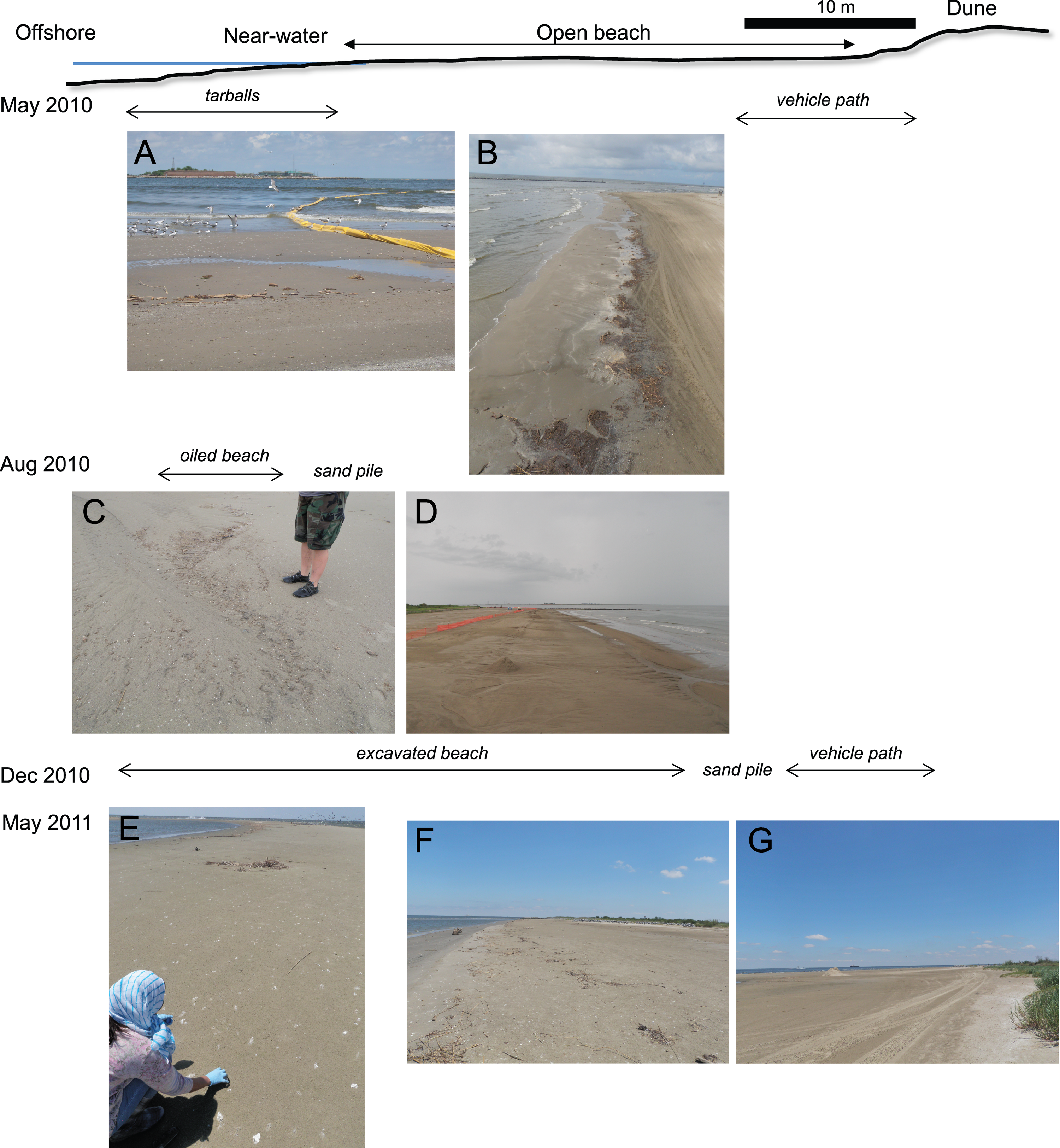

Supplement: Figure S2 — Photographs from specific sampling locations at Grand Isle, Louisiana, with schematic of beach profile and remediation activities shown over time. Generalized remediation activities are also shown on Figure 4. From May 2010, (A) sampling location in the foreshore and swash zone, and (B) looking west along shoreline, with dark areas being tar balls and oceanic debris. From August 2010, (C) tar ball mat in foreshore area of the beach where there was also oil onshore, and (D) looking east along the beach of raked and tilled sand, and piles of sand. There was no sample collection December 2010 because the beach was excavated and there were extensive piles of sand on the open beach. From May 2011, (E) sampling the foreshore, (F) looking west along the beach on open beach, and (G) looking west at the backshore beach where there was still a vehicle path along dune face. All photographs were taken by A.S.E. (TIF) [file pone.0102934.s002.tif]

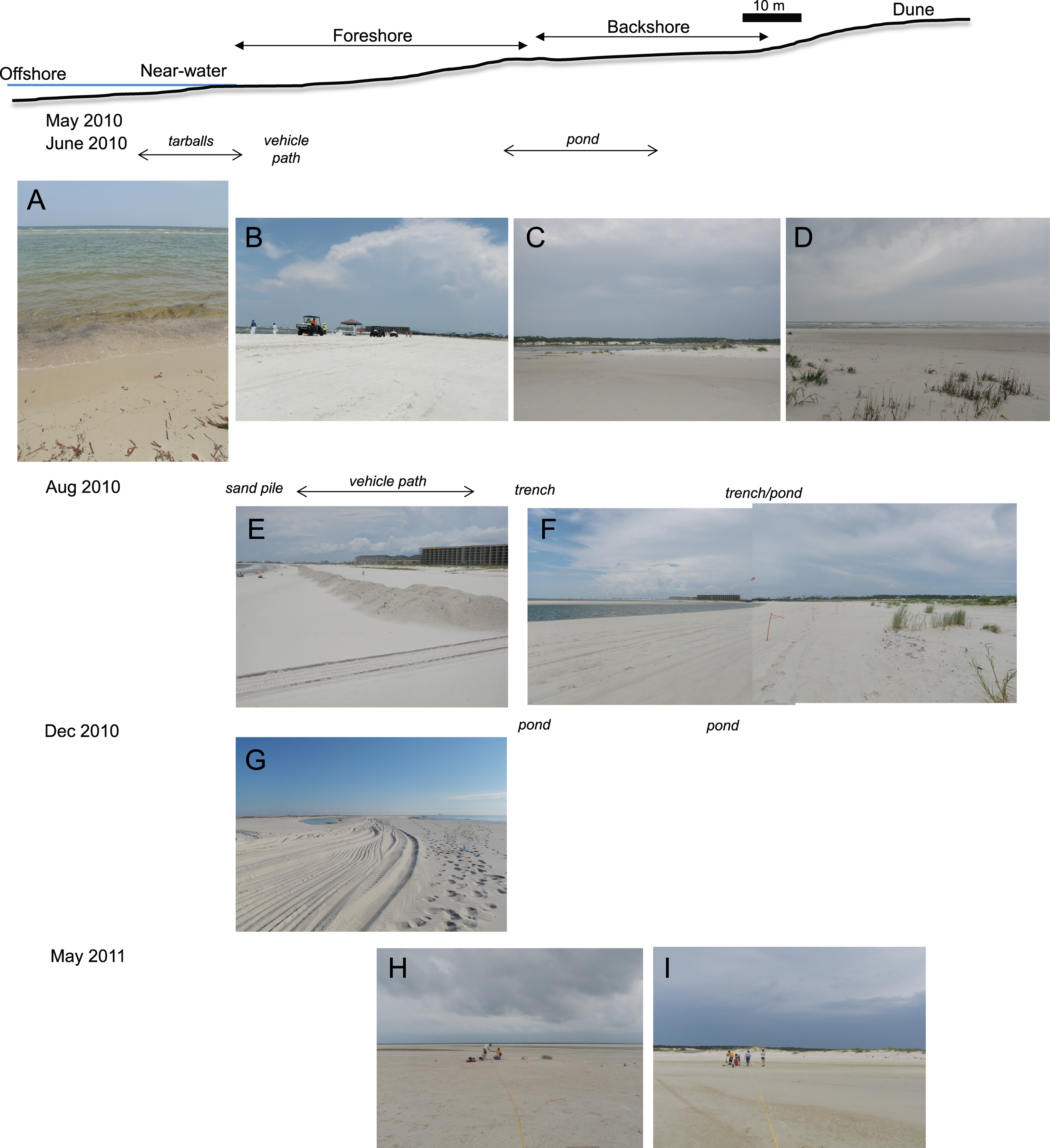

Supplement: Figure S3 — Photographs from specific sampling locations at Dauphin Island, Alabama, with schematic of beach profile and remediation activities shown over time. Generalized remediation activities are also shown on Figure 4. From May and June 2010, (A) looking south from the swash zone, which had tar balls floating in the water and washing up onshore, (B) foreshore area with vehicle path and remediation crew, (C) looking northeast at the backshore of the open beach, showing pond and dunes, and (D) looking south toward the shoreline from the dunes. From August 2010 sampling, (E) large piles of sand were put along the foreshore open beach, behind which was a wide vehicle path, and (F) looking west down the beach from the backshore at deep vehicle tracks. From December 2010 sampling, (G) a vehicle path with deep tracks along the foreshore area comparable to the location of photograph B. From May 2011, (H) looking south from the backshore to the foreshore open beach were there were longer vehicle tracks, and (I) looking north toward the dunes at the open beach. All photographs were taken by A.S.E. (TIF) [file pone.0102934.s003.tif]

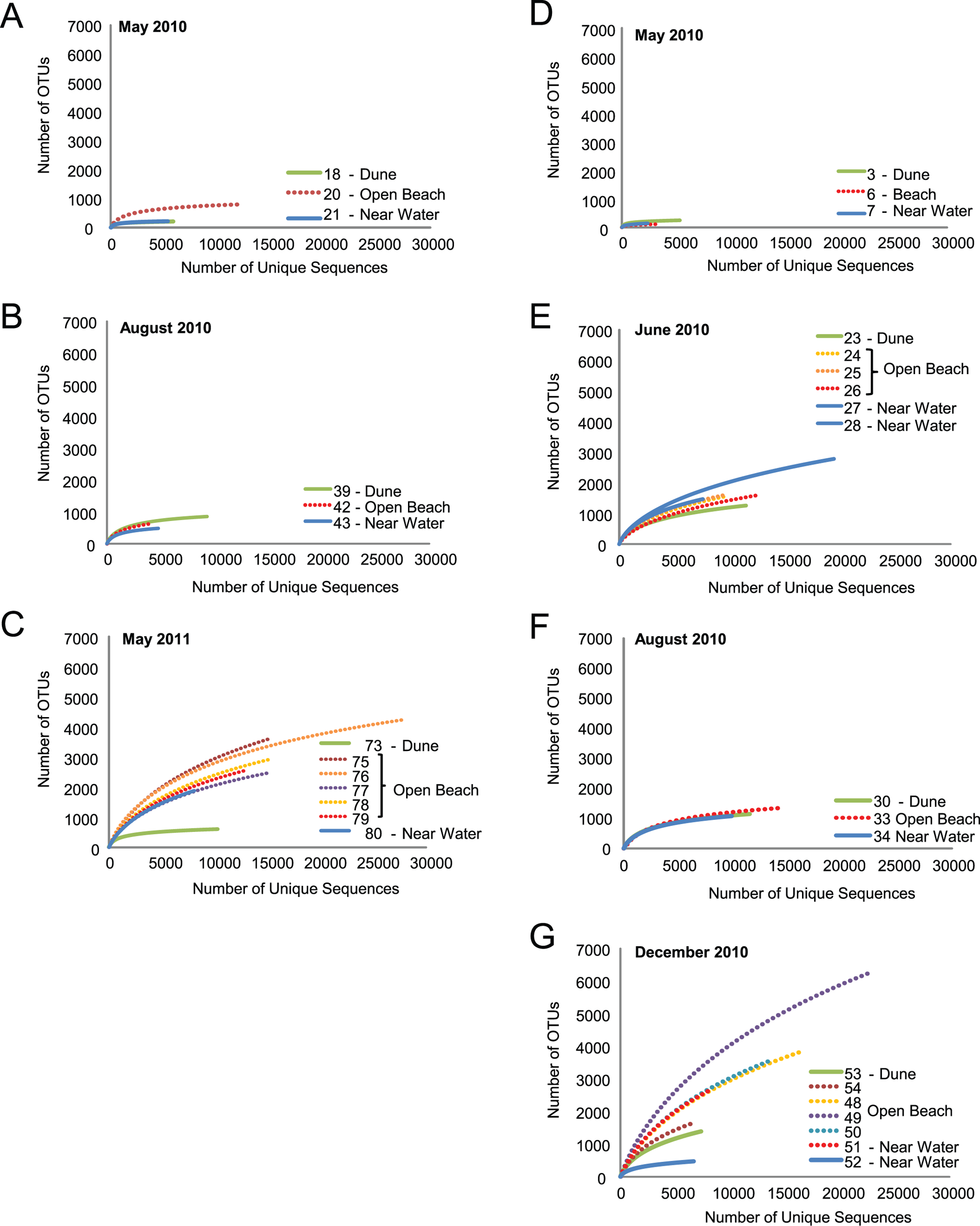

Supplement: Figure S4 — Rarefaction curves for sediment samples from Grand Isle and Dauphin Island. (A) – (C), Grand Isle samples, and (D) – (G) for Dauphin Island samples, summarized for different areas of the beach profiles and all depths for each sampling time. The number of OTUs corresponds to 96% sequence similarity clusters. Refer to text for more pyrosequence processing information. (TIF) [file pone.0102934.s004.tif]

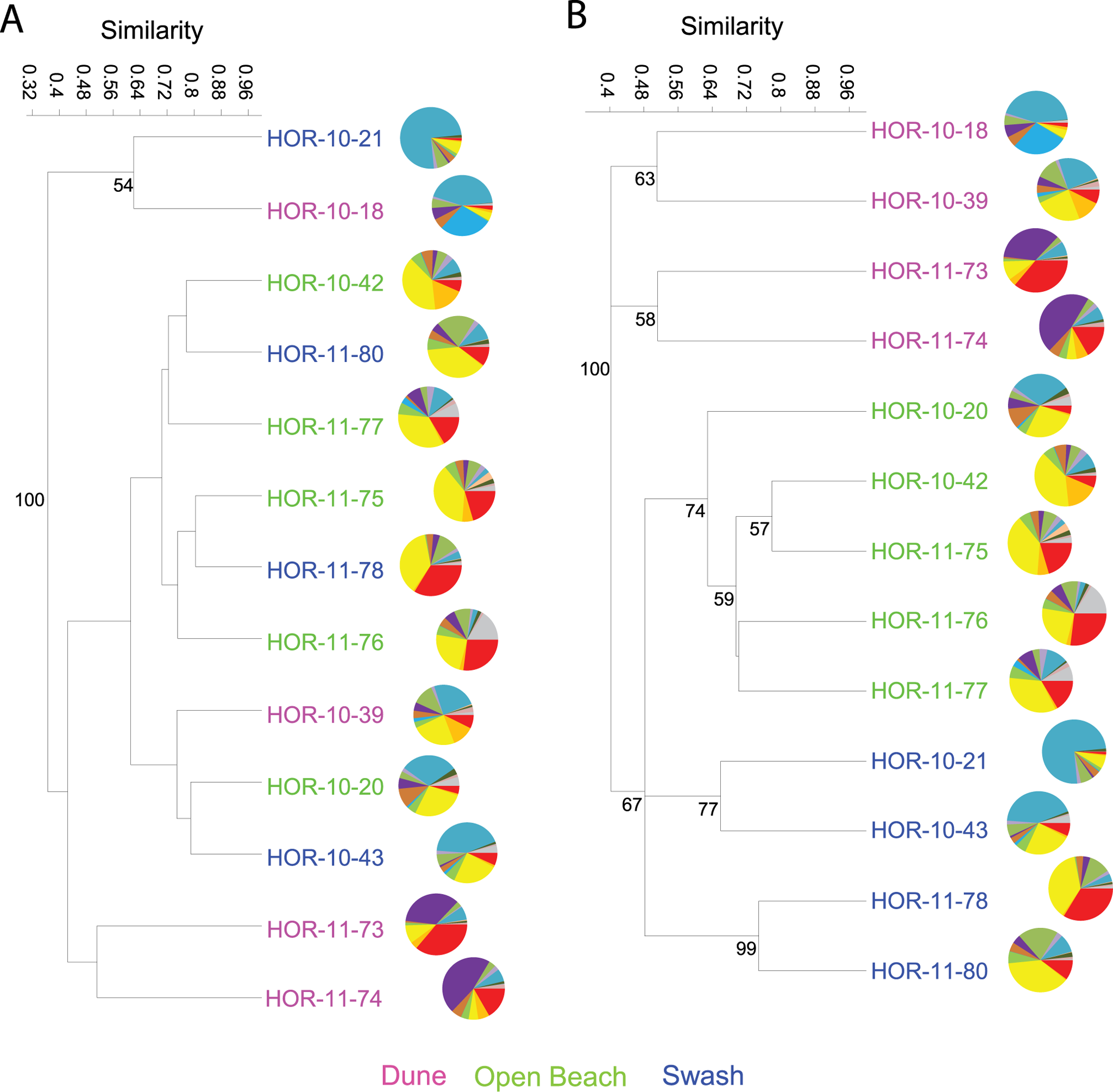

Supplement: Figure S5 — Dendrograms for unweighted pair-group average (UPGMA) hierarchical clustering, or average linkage clustering for sediment communities from Grand Isle samples. (A) UPGMA clustering, or average linkage clustering, constrained by sampling time, from June 2010– May 2011, and (B) UPGMA clustering constrained by location from sampling transects. Clustering was done to evaluate if samples from the same places along the beach, and adjacent to each other, would be more similar. For both dendrograms, clustering was based on Bray-Curtis dissimilarities from normalized abundance data for each taxonomic group, and bootstrap values (in %) for 1000 replicates are given at the nodes (>50%). The cophenetic correlation was 0.8787 for (A) and 0.4902 for (B), suggesting that constraining the cluster analysis to sample location does not yield strong similarities because processes acting on bacterial communities at a particular location may be more similar in adjacent locations than to changes occurring within that location at a particular time. Pie charts for each sample location correspond to Figure 4A. (TIF) [file pone.0102934.s005.tif]

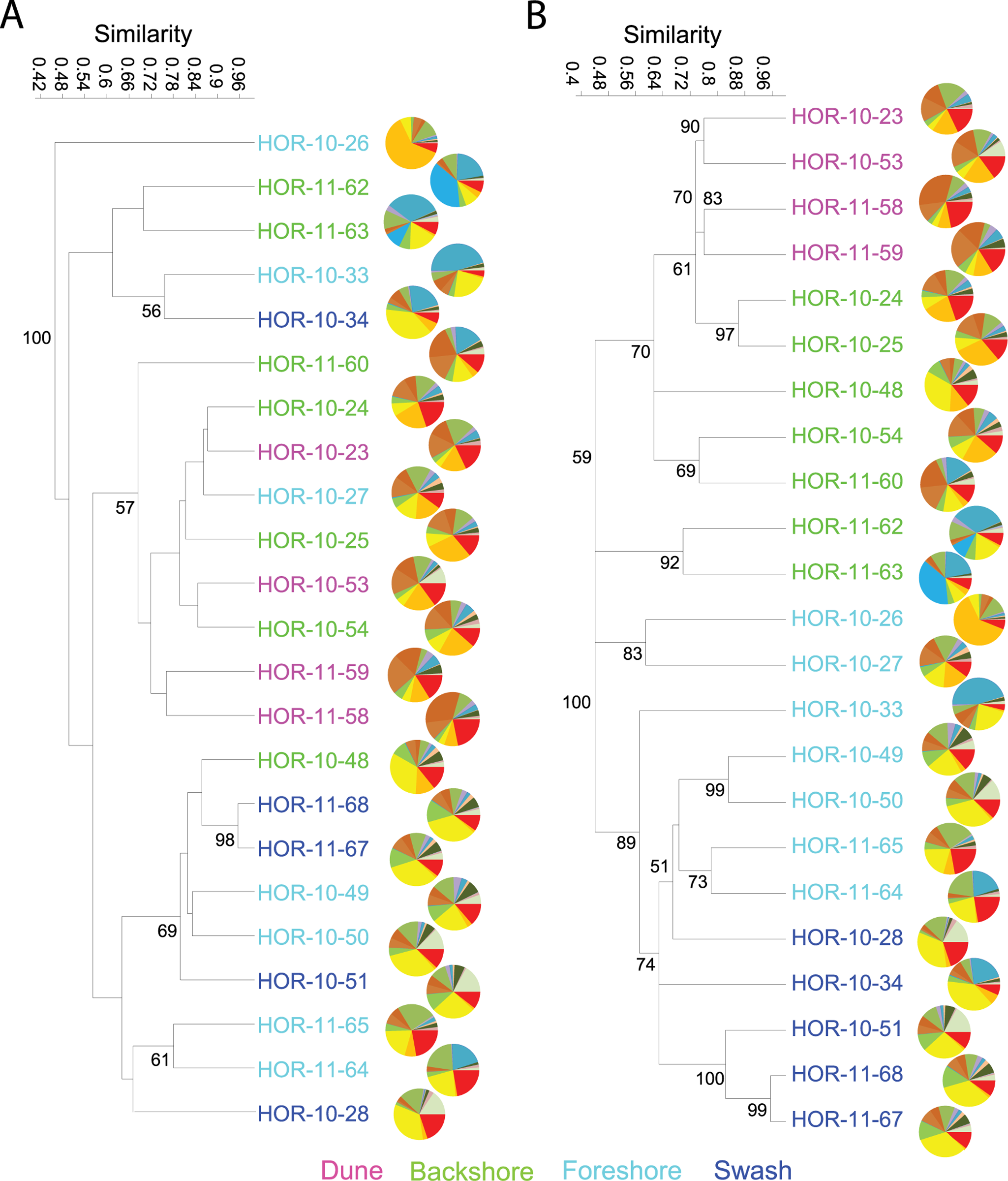

Supplement: Figure S6 — Dendrograms for unweighted pair-group average (UPGMA) hierarchical clustering, or average linkage clustering for sediment communities Dauphin Island samples. (A) UPGMA clustering, or average linkage clustering, constrained by sampling time, from June 2010– May 2011, and (B) UPGMA clustering constrained by location from sampling transects. Clustering was done to evaluate if samples from the same places along the beach, and adjacent to each other, would be more similar. For both dendrograms, clustering was based on Bray-Curtis dissimilarities from normalized abundance data for each taxonomic group, and bootstrap values (in %) for 1000 replicates are given at the nodes (>50%). The cophenetic correlation was 0.8338 for (A) and 0.5885 for (B), suggesting that constraining the cluster analysis to sample location does not yield strong similarities because processes acting on bacterial communities at a particular location may be more similar in adjacent locations than to changes occurring within that location at a particular time. Pie charts for each sample location correspond to Figure 4B. (TIF) [file pone.0102934.s006.tif]

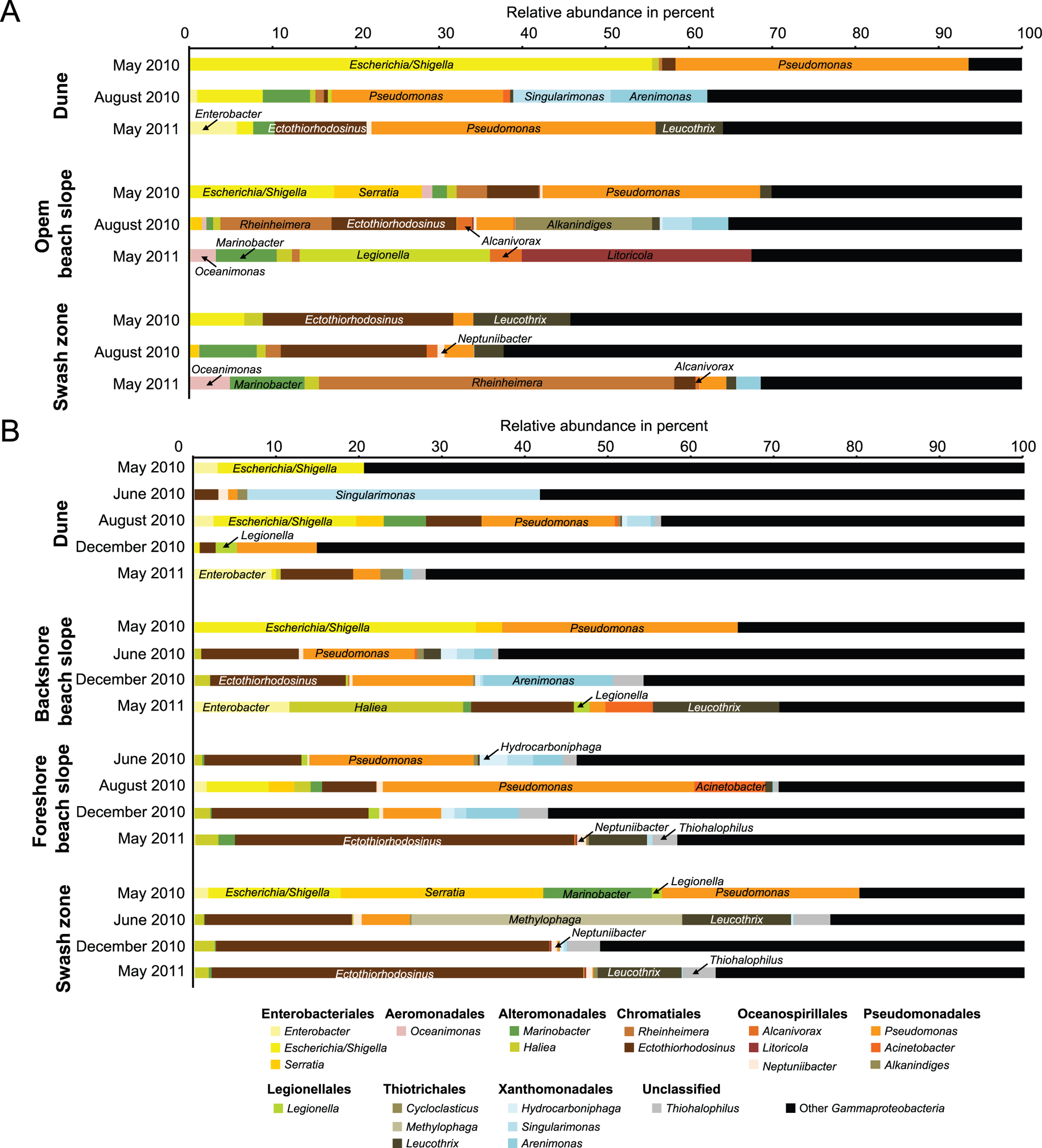

Supplement: Figure S7 — Genus-level taxonomic results for Gammaproteobacteria . Summaries are organized by sampling time and by beach location for (A) Grand Isle and (B) Dauphin Island. (TIF) [file pone.0102934.s007.tif]
