# Supplementary material for: Regime Shift in Sandy Beach Microbial Communities following Deepwater Horizon Oil Spill Remediation Efforts
Source: PLoS One. 2014 Jul 18;9(7):e102934. doi: 10.1371/journal.pone.0102934 (PMC4103866; doi:10.1371/journal.pone.0102934)
Supplement: Table S1 — Location and physicochemical data for each of the sediment samples collected from Grand Isle, Louisiana, and Dauphin Island, Alabama, 2010–2011. Grand Isle samples are shaded in gray. N.M. = not measured. (PDF) [file pone.0102934.s008.pdf]

**Table S1**. **Location and physicochemical data for each of the sediment samples collected from Grand Isle, Louisiana, and Dauphin Island, Alabama, 2010-2011.** Grand Isle samples are shaded in gray. N.M. = not measured.

| Sample Code | Latitude | Longitude | Sampling Date | Sediment pH | Average Water Content (%) (± S.D.) | Average Organic Carbon Content (%) (± S.D.) |
|---|---|---|---|---|---|---|
| HOR-10-03A | 30$^o$ 14’ 40.1” | 88$^o$ 07’ 27.5” | 05-02-10 | 8.22 | 2.86 (0.03) | 2.82 (0.01) |
| HOR-10-03B | | | | | | |
| HOR-10-03C | | | | | | |
| HOR-10-06A | 30$^o$ 14’ 40.1” | 88$^o$ 07’ 29.0” | 05-02-10 | 6.08 | 12.63 (0.04) | 0.39 (0.01) |
| HOR-10-06B | | | | | 18.68 (0.08) | 0.18 (0.004) |
| HOR-10-07B | 30$^o$ 14’ 39.9” | 88$^o$ 07’ 29.5” | 05-02-10 | 6.26 | 6.64 | 0.11 |
| HOR-10-18A | 29$^o$ 16' 05.8" | 89$^o$ 57' 08.6" | 05-22-10 | 8.36 | 2.56 (0.77) | 0.16 (0.02) |
| HOR-10-18B | | | | 8.62 | 4.50 (0.25) | 0.34 (0.005) |
| HOR-10-20A | 29$^o$ 16' 06.2" | 89$^o$ 57' 07.8" | 05-22-10 | 7.33 | 17.26 (0.37) | 0.32 (0.003) |
| HOR-10-20B | | | | 7.57 | 21.88 (0.46) | 0.93 (0.005) |
| HOR-10-21B | 29$^o$ 16' 06.1" | 89$^o$ 57' 07.8" | 05-22-10 | 7.60 | 18.79 (0.39) | 0.07 (0.05) |
| HOR-10-23A | 30$^o$ 14’ 39.6” | 88$^o$ 07’ 27.1” | 06-01-10 | 6.7 | 2.19 (0.31) | 0.07 (0.002) |
| HOR-10-23B | | | | 8.38 | 4.89 (0.79) | 0.04 (0.005) |
| HOR-10-23C | | | | 7.91 | 4.72 (0.52) | 0.04 (0.01) |
| HOR-10-24A | 30$^o$ 14’ 39.5” | 88$^o$ 07’ 27.5” | 06-01-10 | 8.55 | 0.07 (0.04) | 0.21 (0.25) |
| HOR-10-24B | | | | 6.37 | 4.95 (0.68) | 0.03 (0.01) |
| HOR-10-24C | | | | 7.03 | 3.29 (0.56) | 0.04 (0.05) |
| HOR-10-25A | 30$^o$ 14’ 39.4” | 88$^o$ 07’ 28.1” | 06-01-10 | 7.63 | 0.03 (0.00) | 0.02 (0.01) |
| HOR-10-25B | | | | 7.59 | 4.45 (0.17) | 0.01 (0.01) |
| HOR-10-25C | | | | 8.24 | 7.25 (0.15) | 0.02 (0.003) |
| HOR-10-26A | 30$^o$ 14’ 39.2” | 88$^o$ 07’ 28.6” | 06-01-10 | 9.04 | 0.02 (0.00) | 0.03 (0.02) |
| HOR-10-26B | | | | 7.70 | 2.80 (0.50) | 0.02 (0.004) |
| HOR-10-26C | | | | 7.41 | 4.35 (0.34) | 0.02 (0.007) |
| HOR-10-27A | 30$^o$ 14’ 39.1” | 88$^o$ 07’ 29.1” | 06-01-10 | 7.86 | 0.12 (0.08) | 0.04 (0.01) |
| HOR-10-27B | | | | 8.27 | 4.51 (0.64) | 0.04 (0.001) |
| HOR-10-27C | | | | 8.71 | 4.23 (0.36) | 0.04 (0.01) |
| HOR-10-28A | 30$^o$ 14’ 39.1” | 88$^o$ 07’ 29.3” | 06-01-10 | 8.08 | 9.18 (0.12) | 0.11 (0.01) |
| HOR-10-28B | | | | 8.40 | 12.94 (0.61) | 0.10 (0.01) |
| HOR-10-28C | | | | 7.90 | 15.56 (0.22) | 0.11 (0.005) |
| HOR-10-28oil | 30$^o$ 14’ 39.1” | 88$^o$ 07’ 29.3” | 06-01-10 | N.M. | 14.61 (0.45) | 1.66 (0.87) |

| Sample Code | Latitude | Longitude | Sampling Date | Sediment pH | Average Water Content (%) (± S.D.) | Average Organic Carbon Content (%) (± S.D.) |
|---|---|---|---|---|---|---|
| HOR-10-30A | 30° 14’ 39.2” | 88° 07’ 27.25” | 08-14-10 | 7.37 | 0.99 (0.51) | 0.03 (0.01) |
| HOR-10-30B | | | | 7.65 | 4.12 (0.41) | 0.03 (0.01) |
| HOR-10-30C | | | | 7.43 | 6.52 (0.06) | 0.02 (0.004) |
| HOR-10-33A | 30° 14’ 38.56” | 88° 07’ 29.39” | 08-14-10 | 6.69 | 4.88 (0.1) | 0.51 (0.01) |
| HOR-10-33B | | | | 7.85 | 2.52 (0.18) | 0.18 (0.001) |
| HOR-10-33C | | | | 7.23 | 3.30 (0.11) | 0.32 (0.06) |
| HOR-10-34A | 30° 14’ 38.46” | 88° 07’ 29.6” | 08-14-10 | 6.99 | 0.35 (0.08) | 0.18 (0.06) |
| HOR-10-34B | | | | 6.57 | 14.73 (0.19) | 0.31 (0.41) |
| HOR-10-34C | | | | 6.89 | 3.20 (0.09) | 0.05 (0.02) |
| HOR-10-39A | 29° 16' 08.5" | 89° 57' 12.4" | 08-15-10 | 7.90 | 13.00 (0.25) | 0.25 (0.03) |
| HOR-10-39B | | | | 8.30 | 13.00 (0.17) | 0.17 (0.01) |
| HOR-10-42A | 29° 16' 09.8" | 89° 57' 12.3" | 08-15-10 | 8.18 | 14.29 (0.09) | 0.20 (0.04) |
| HOR-10-42B | | | | 7.89 | 16.58 (1.13) | 0.82 (0.49) |
| HOR-10-43A | 29° 16' 10.4" | 89° 57' 11.2" | 08-15-10 | 7.89 | 16.94 (0.08) | 0.28 (0.13) |
| HOR-10-53A | 30° 14’ 40.4” | 88° 07’ 27.1” | 12-10-10 | 7.49 | 0.05 (0.03) | 0.02 (0.001) |
| HOR-10-53B | | | | 7.43 | 0.83 (0.11) | 0.01 (0.002) |
| HOR-10-53C | | | | 7.10 | 2.21 | 0.05 |
| HOR-10-54A | 30° 14’ 40.1” | 88° 07’ 27.9” | 12-10-10 | 7.62 | 0.04 (0.02) | 0.04 (0.004) |
| HOR-10-54B | | | | 7.35 | 2.05 (0.56) | 0.06 (0.05) |
| HOR-10-54C | | | | 7.21 | 2.33 (0.39) | 0.04 (0.02) |
| HOR-10-48A | 30° 14’ 39.9” | 88° 07’ 30.9” | 12-10-10 | 9.03 | 2.62 (0.44) | 0.57 (0.02) |
| HOR-10-48B | | | | 8.07 | 2.91 (0.06) | 1.27 (0.009) |
| HOR-10-48C | | | | 8.22 | 5.81 (0.1) | 1.34 (0.07) |
| HOR-10-49A | 30° 14’ 39.1” | 88° 07’ 32.6” | 12-10-10 | 7.04 | 0.04 (0.003) | 0.04 (0.006) |
| HOR-10-49B | | | | 7.92 | 4.09 (0.1) | 0.1 (0.005) |
| HOR-10-49C | | | | 7.31 | 6.97 (0.07) | 0.93 (0.009) |
| HOR-10-50A | 30° 14’ 38.5” | 88° 07’ 33.6” | 12-10-10 | 7.37 | 0.04 (0.05) | 0.02 (0.002) |
| HOR-10-50B | | | | 6.80 | 3.74 (0.04) | 0.23 (0.01) |
| HOR-10-50C | | | | 7.33 | 6.06 | 0.04 |
| HOR-10-51A | 30° 14’ 38.4” | 88° 07’ 33.9” | 12-10-10 | 7.76 | 17.56 (0.37) | 0.11 (0.002) |
| HOR-10-51B | | | | 7.57 | 15.55 | 0.07 |
| HOR-10-52A | 30° 14’ 38.2” | 88° 07’ 34.0” | | 7.38 | 17.48 (0.24) | 0.13 (0.005) |
| HOR-11-73A | 29° 16' 09.3" | 89° 57' 13.4" | 05-14-11 | 7.17 | 0.04 (0.01) | 0.34 (0.02) |
| HOR-11-73B | | | | 7.50 | 0.21 (0.003) | 0.36 (0.009) |
| HOR-11-73C | | | | 7.49 | 0.21 (0.01) | 0.33 (0.02) |

| Sample Code | Latitude | Longitude | Sampling Date | Sediment pH | Average Water Content (%) (± S.D.) | Average Organic Carbon Content (%) (± S.D.) |
|---|---|---|---|---|---|---|
| HOR-11-74A | 29° 16' 09.4" | 89° 57' 13.7" | 05-14-11 | 7.32 | 0.07 (0.008) | 0.48 (0.04) |
| HOR-11-74B | | | | 7.99 | 1.19 (0.72) | 0.24 (0.02) |
| HOR-11-74C | | | | 7.95 | 1.82 (0.77) | 0.24 (0.002) |
| HOR-11-75A | 29° 16' 09.6" | 89° 57' 13.6" | 05-14-11 | 8.85 | 5.98 (0.08) | 0.52 (0.04) |
| HOR-11-75B | | | | 8.18 | 15.37 (0.18) | 0.35 (0.03) |
| HOR-11-75C | | | | 8.11 | 15.50 (1.02) | 0.32 (0.03) |
| HOR-11-76A | 29° 16' 09.9" | 89° 57' 13.0" | 05-14-11 | 7.50 | 9.85 (0.52) | 0.79 (0.02) |
| HOR-11-76B | | | | 7.71 | 13.92 (0.59) | 0.26 (0.10) |
| HOR-11-76C | | | | 7.44 | 13.65 (0.42) | 0.59 (0.65) |
| HOR-11-77A | 29° 16' 10.0" | 89° 57' 11.1" | 05-14-11 | 7.48 | 11.82 (0.15) | 1.51 (0.19) |
| HOR-11-77B | | | | 7.59 | 16.07 (0.85) | 0.61 (0.03) |
| HOR-11-77C | | | | 7.68 | 14.98 (0.29) | 0.57 (0.03) |
| HOR-11-78A | 29° 16' 10.3" | 89° 57' 10.7" | 05-14-11 | 7.33 | 6.08 (0.04) | 1.14 (0.08) |
| HOR-11-78B | | | | 7.63 | 13.79 (0.49) | 0.30 (0.006) |
| HOR-11-78C | | | | 7.81 | 15.10 (0.19) | 0.39 (0.07) |
| HOR-11-79A | 29° 16' 10.3" | 89° 57' 10.1" | 05-14-11 | 7.20 | 10.98 (0.15) | 0.24 (0.02) |
| HOR-11-79B | | | | 7.12 | 16.40 (0.20) | 0.23 (0.005) |
| HOR-11-79C | | | | 7.07 | 22.61 (8.10) | 0.21 (0.008) |
| HOR-11-80A | 29° 16' 10.4" | 89° 57' 09.9" | 05-14-11 | 7.43 | 34.81 (8.46) | 0.30 (0.05) |
| HOR-11-80B | | | | 7.49 | 15.58 (0.53) | 0.23 (0.02) |
| HOR-11-58A | 30° 14’ 35.5” | 88° 07’ 25.6” | 05-13-11 | 6.84 | 0.04 (0.01) | 0.02 (0.005) |
| HOR-11-58B | | | | 6.78 | 11.99 (1.16) | 0.02 (0.008) |
| HOR-11-58C | | | | 7.02 | 0.07 (0.01) | 0.02 (0.001) |
| HOR-11-59A | 30° 14’ 35.3” | 88° 07’ 26.1” | 05-13-11 | 6.76 | 0.05 (0.007) | 0.02 (0.005) |
| HOR-11-59B | | | | 6.28 | 2.34 (0.52) | 0.01 (0.007) |
| HOR-11-59C | | | | 6.36 | 2.56 (2.16) | 0.02 (0.007) |
| HOR-11-60A | 30° 14’ 34.9” | 88° 07’ 27.1” | 05-13-11 | 6.95 | 0.06 (0.004) | 0.05 (0.06) |
| HOR-11-60B | | | | 6.60 | 3.16 (0.63) | 0.03 (0.004) |
| HOR-11-60C | | | | 6.37 | 4.17 (0.09) | 0.03 (0.005) |
| HOR-11-62A | 30° 14’ 34.5” | 88° 07’ 27.7” | 05-13-11 | 8.62 | 11.63 (0.17) | 0.12 (0.03) |
| HOR-11-62B | | | | 7.96 | 15.08 (0.71) | 0.13 (0.04) |
| HOR-11-62C | | | | 7.48 | 14.72 (0.41) | 0.11 (0.04) |
| HOR-11-63A | 30° 14’ 34.1” | 88° 07’ 28.4” | 05-13-11 | 8.58 | 11.06 (0.02) | 0.08 (0.01) |
| HOR-11-63B | | | | 7.80 | 15.61 (0.22) | 0.14 (0.01) |
| HOR-11-63C | | | | 7.43 | 17.59 (1.04) | 0.15 (0.01) |

| Sample Code | Latitude | Longitude | Sampling Date | Sediment pH | Average Water Content (%) (± S.D.) | Average Organic Carbon Content (%) (± S.D.) |
|---|---|---|---|---|---|---|
| HOR-11-64A | $30^o$ 14’ 32.8” | $88^o$ 07’ 30.5” | 05-13-11 | 7.74 | 14.41 (0.35) | 0.15 (0.01) |
| HOR-11-64B | | | | 7.62 | 16.20 (0.38) | 0.18 (0.01) |
| HOR-11-64C | | | | 7.59 | 16.65 (0.38) | 0.15 (0.007) |
| HOR-11-65A | $30^o$ 14’ 32.7” | $88^o$ 07’ 31.2” | 05-13-11 | 7.71 | 5.03 (0.85) | 0.04 (0.00) |
| HOR-11-65B | | | | 7.33 | 5.68 (0.13) | 0.05 (0.002) |
| HOR-11-65C | | | | 7.60 | 8.69 (0.31) | 0.07 (0.004) |
| HOR-11-67A | $30^o$ 14’ 32.2” | $88^o$ 07’ 32.3” | 05-13-11 | 7.45 | 6.63 (0.32) | 0.04 (0.003) |
| HOR-11-67B | | | | 7.26 | 11.10 (0.26) | 0.05 (0.006) |
| HOR-11-67C | | | | 7.34 | 13.21 (0.56) | 0.07 (0.003) |
| HOR-11-68A | $30^o$ 14’ 32.1” | $88^o$ 07’ 32.5” | 05-13-11 | 7.34 | 20.75 (0.34) | 0.09 (0.006) |
| HOR-11-68B | | | | 7.75 | 14.40 (0.51) | 0.09 (0.01) |
| HOR-11-68T | | | | 7.41 | 6.33 (0.10) | 6.77 (0.06) |
