# Supplementary material for: Regime Shift in Sandy Beach Microbial Communities following Deepwater Horizon Oil Spill Remediation Efforts
Source: PLoS One. 2014 Jul 18;9(7):e102934. doi: 10.1371/journal.pone.0102934 (PMC4103866; doi:10.1371/journal.pone.0102934)
Supplement: Table S2 — Summary of pyrosequencing data for each of the sediment samples collected from Grand Isle, Louisiana, and Dauphin Island, Alabama, 2010–2011. Grand Isle samples are shaded in gray. Operational Taxonomic Units (OTUs) and diversity indices were calculated using MOTHUR. (PDF) [file pone.0102934.s009.pdf]

Table S2. **Summary of pyrosequencing data for each of the sediment samples collected from Grand Isle, Louisiana, and Dauphin Island, Alabama, 2010-2011.** Grand Isle samples are shaded in gray. Operational Taxonomic Units (OTUs) and diversity indices were calculated using MOTHUR.

Sample Code	Small Read Archive Run Accession Number	Number of Seqs. (raw)	Number of Seqs. After Trimming	Average Seq. Length After Trimming	Chimeric Seq.	% Chimeric	OTUs (96% sequence identity)	Chao1 Index	Shannon Index	Simpson Index
HOR-10-03A	SRR628470	2306	1891	389.4	90	4.76%	71	82.67	3.61	0.04
HOR-10-03B	SRR628471	4004	3231	396.8	299	9.25%	114	137.75	3.93	0.04
HOR-10-03C	SRR628472	2039	1590	385.0	63	3.96%	91	128.80	3.60	0.05
HOR-10-06A	SRR628473	3781	3102	384.2	33	1.06%	94	121.60	3.56	0.05
HOR-10-06B	SRR628474	894	736	383.4	16	2.17%	28	40.00	2.50	0.11
HOR-10-07B	SRR628475	4132	3122	394.1	278	8.90%	108	121.64	3.53	0.06
HOR-10-18A	SRR628476	1982	1439	386.9	39	2.71%	48	59.00	3.03	0.07
HOR-10-18B	SRR628477	6777	5626	381.0	147	2.61%	158	204.43	4.08	0.03
HOR-10-20A	SRR628478	10703	8722	383.5	358	4.10%	530	632.51	5.13	0.01
HOR-10-20B	SRR628479	6635	5441	408.1	138	2.54%	371	409.13	5.18	0.01
HOR-10-21B	SRR628480	11013	7885	359.7	56	0.71%	218	245.05	3.98	0.06
HOR-10-23A	SRR628481	7270	5080	333.5	131	2.58%	823	1198.47	5.86	0.01
HOR-10-23B	SRR628482	5491	3631	387.0	85	2.34%	647	993.26	5.64	0.01
HOR-10-23C	SRR628483	13145	5270	354.6	157	2.98%	378	511.47	4.70	0.02
HOR-10-24A	SRR628484	5739	2689	370.2	93	3.46%	459	726.12	5.34	0.01
HOR-10-24B	SRR628485	13442	4290	374.9	79	1.84%	527	815.92	4.97	0.03
HOR-10-24C	SRR628486	17047	5200	359.2	63	1.21%	997	1670.39	5.90	0.01
HOR-10-25A	SRR628487	15158	5919	364.2	90	1.52%	1084	1845.88	5.90	0.01
HOR-10-25B	SRR628488	11312	5265	371.3	45	0.85%	501	727.45	4.94	0.02
HOR-10-25C	SRR628489	2511	1299	366.0	7	0.54%	416	954.39	5.14	0.02
HOR-10-26A	SRR628490	14575	7798	372.8	52	0.67%	956	1947.09	5.12	0.04
HOR-10-26B	SRR628491	9783	5256	354.4	72	1.37%	293	368.31	4.34	0.03
HOR-10-26C	SRR628492	17116	9631	371.8	82	0.85%	706	1175.81	4.48	0.07
HOR-10-27A	SRR628493	14526	6896	371.1	51	0.74%	1140	1694.96	6.16	0.00
HOR-10-27B	SRR628494	2458	1168	358.3	30	2.57%	208	503.04	4.47	0.02
HOR-10-27C	SRR628495	2825	1256	362.1	7	0.56%	348	742.83	5.14	0.01
HOR-10-28A	SRR628496	13193	6670	306.5	1215	18.22%	1521	2547.23	6.20	0.01
HOR-10-28B	SRR628497	13193	6670	331.1	179	2.68%	1520	2547.11	6.19	0.01
HOR-10-28C	SRR628498	12352	9192	327.5	574	6.24%	782	1126.98	5.47	0.01
HOR-10-28oil	SRR628499	6263	1710	352.2	96	5.61%	252	500.11	4.45	0.02
HOR-10-30A	SRR628500	9505	7780	386.4	201	2.58%	884	1133.27	5.81	0.01

Sample Code	Small Read Archive Run Accession Number	Number of Seqs. (raw)	Number of Seqs. After Trimming	Average Seq. Length After Trimming	Chimeric Seq.	% Chimeric	OTUs (96% sequence identity)	Chao1 Index	Shannon Index	Simpson Index
HOR-10-30B	SRR628501	3560	2876	397.0	66	2.29%	279	340.42	4.91	0.01
HOR-10-30C	SRR628502	3517	2850	407.4	137	4.81%	125	160.43	3.43	0.08
HOR-10-33A	SRR628503	9311	7728	374.8	452	5.85%	360	447.42	4.39	0.03
HOR-10-33B	SRR628504	5780	4749	388.1	132	2.78%	158	193.20	4.06	0.03
HOR-10-33C	SRR628505	6307	5189	405.8	135	2.60%	962	1265.47	6.35	0.00
HOR-10-34A	SRR628506	4537	3543	383.4	136	3.84%	151	189.15	4.26	0.02
HOR-10-34B	SRR628507	5843	4847	402.9	154	3.18%	744	907.43	5.95	0.00
HOR-10-34C	SRR628508	4623	3824	408.6	62	1.62%	313	418.11	3.81	0.10
HOR-10-39A	SRR628509	6054	4956	389.5	149	3.01%	392	455.61	5.15	0.01
HOR-10-39B	SRR628510	7001	5577	396.0	114	2.04%	556	635.44	5.53	0.01
HOR-10-42A	SRR628511	5629	4686	397.3	168	3.59%	614	787.79	5.53	0.01
HOR-10-42B	SRR628512	836	79	386.7	3	3.80%	27	65.25	2.79	0.08
HOR-10-43A	SRR628513	6788	5635	405.3	93	1.65%	482	575.64	4.90	0.03
HOR-10-53A	SRR628526	8043	5204	294.5	144	2.77%	814	1190.04	5.86	0.01
HOR-10-53B	SRR628527	2906	1764	290.9	29	1.64%	349	544.15	5.03	0.02
HOR-10-53C	SRR628528	3421	2160	292.9	61	2.82%	481	912.06	5.29	0.01
HOR-10-54A	SRR628529	3749	2376	293.7	46	1.94%	617	1183.16	5.79	0.01
HOR-10-54B	SRR628530	2918	1837	289.8	44	2.40%	400	637.54	5.31	0.01
HOR-10-54C	SRR628531	6290	4145	297.7	41	0.99%	940	1447.61	6.09	0.00
HOR-10-48A	SRR628514	14750	10375	337.5	419	4.04%	2322	3869.25	6.87	0.00
HOR-10-48B	SRR628515	8343	6205	338.5	520	8.38%	1614	3075.47	6.47	0.00
HOR-10-48C	SRR628516	4945	3093	373.9	130	4.20%	885	1957.39	5.81	0.01
HOR-10-49A	SRR628517	12461	5208	367.4	102	1.96%	1450	2577.56	6.57	0.00
HOR-10-49B	SRR628518	7136	5095	338.5	196	3.85%	2022	3900.76	7.15	0.00
HOR-10-49C	SRR628519	15550	9420	343.7	433	4.60%	3040	5419.02	7.45	0.00
HOR-10-50A	SRR628520	7143	4052	343.7	111	2.74%	1450	2368.99	6.76	0.00
HOR-10-50B	SRR628521	9373	6743	331.5	280	4.15%	1371	2476.81	5.96	0.01
HOR-10-50C	SRR628522	9018	4995	342.8	191	3.82%	1877	3623.76	7.02	0.00
HOR-10-51A	SRR628523	8818	6464	341.6	153	2.37%	2051	3662.44	6.81	0.00
HOR-10-51B	SRR628524	4563	2902	286.1	69	2.38%	1201	2911.32	6.58	0.00
HOR-10-52A	SRR628525	12642	9195	342.1	245	2.66%	473	689.44	4.72	0.02
HOR-11-73A	SRR628559	8303	6084	352.1	183	3.01%	233	258.63	4.59	0.02
HOR-11-73B	SRR628560	4669	3347	360.1	112	3.35%	214	235.58	4.73	0.01
HOR-11-73C	SRR628561	4234	3001	343.2	41	1.37%	247	296.88	4.83	0.01
HOR-11-74A	SRR628562	4131	3106	358.0	168	5.41%	292	362.22	4.71	0.02
HOR-11-74B	SRR628563	4258	3153	363.8	53	1.68%	362	463.13	5.18	0.01

Sample Code	Small Read Archive Run Accession Number	Number of Seqs. (raw)	Number of Seqs. After Trimming	Average Seq. Length After Trimming	Chimeric Seq.	% Chimeric	OTUs (96% sequence identity)	Chao1 Index	Shannon Index	Simpson Index
HOR-11-74C	SRR628564	11980	8795	356.7	187	2.13%	419	523.07	5.22	0.01
HOR-11-75A	SRR628565	10258	7692	343.9	575	7.48%	616	827.50	5.36	0.01
HOR-11-75B	SRR628566	7792	5660	358.5	148	2.61%	1695	2540.55	6.83	0.00
HOR-11-75C	SRR628567	5759	4117	358.3	186	4.52%	1545	2783.59	6.87	0.00
HOR-11-76A	SRR628568	9603	6846	349.2	347	5.07%	991	1398.82	5.92	0.01
HOR-11-76B	SRR628569	11055	8261	360.6	303	3.67%	482	567.11	5.45	0.01
HOR-11-76C	SRR628570	23770	16999	355.9	721	4.24%	3020	4032.04	7.30	0.00
HOR-11-77A	SRR628571	7193	5586	344.9	229	4.10%	361	494.90	4.85	0.02
HOR-11-77B	SRR628572	9706	7028	347.9	418	5.95%	1296	1913.60	6.08	0.01
HOR-11-77C	SRR628573	9195	6400	342.7	407	6.36%	1298	2124.56	6.13	0.01
HOR-11-78A	SRR628574	10172	7388	353.7	1006	13.62%	875	1179.45	5.75	0.01
HOR-11-78B	SRR628575	7868	5612	348.7	370	6.59%	1396	2190.89	6.42	0.00
HOR-11-78C	SRR628576	8565	5900	343.5	421	7.14%	1221	1921.42	6.12	0.01
HOR-11-79A	SRR628577	9616	6630	341.3	573	8.64%	868	1384.38	5.60	0.01
HOR-11-79B	SRR628578	8201	5275	334.1	323	6.12%	1230	2140.92	6.22	0.01
HOR-11-79C	SRR628579	5987	4050	350.4	275	6.79%	1200	1894.43	6.41	0.00
HOR-11-80A	SRR628580	7051	5014	336.2	299	5.96%	955	1599.92	5.68	0.01
HOR-11-80B	SRR628581	6610	4658	341.9	328	7.04%	1273	2187.52	6.35	0.00
HOR-11-58A	SRR628532	6877	4740	380.6	288	6.08%	939	1377.68	6.11	0.00
HOR-11-58B	SRR628533	6053	4500	382.8	312	6.93%	910	1361.15	6.11	0.00
HOR-11-58C	SRR628534	6726	4977	379.2	268	5.38%	932	1334.42	6.14	0.00
HOR-11-59A	SRR628535	5981	3822	379.0	176	4.60%	816	1151.16	5.97	0.01
HOR-11-59B	SRR628536	6620	4879	393.7	119	2.44%	1172	1801.61	6.35	0.00
HOR-11-59C	SRR628537	7537	5674	389.2	552	9.73%	1155	1718.27	6.17	0.01
HOR-11-60A	SRR628538	7642	5141	386.8	309	6.01%	849	1155.00	5.85	0.01
HOR-11-60B	SRR628539	7240	5388	391.2	110	2.04%	1147	1741.24	6.21	0.01
HOR-11-60C	SRR628540	6566	5138	400.8	217	4.22%	766	1200.47	4.89	0.05
HOR-11-62A	SRR628541	8224	6229	406.5	783	12.57%	468	754.36	4.26	0.04
HOR-11-62B	SRR628542	6667	4799	380.4	653	13.61%	1127	2020.26	6.04	0.01
HOR-11-62C	SRR628543	7275	4698	375.7	394	8.39%	953	1642.66	5.79	0.01
HOR-11-63A	SRR628544	6889	4724	401.9	476	10.08%	342	519.79	3.91	0.09
HOR-11-63B	SRR628545	6709	5224	375.0	458	8.77%	775	1520.28	5.14	0.02
HOR-11-63C	SRR628546	5562	4060	389.1	314	7.73%	543	963.15	4.67	0.03
HOR-11-64A	SRR628547	11637	8518	349.3	952	11.18%	787	1200.19	5.28	0.02
HOR-11-64B	SRR628548	7562	5516	344.7	905	16.41%	1264	2280.46	6.20	0.01
HOR-11-64C	SRR628549	6634	4572	329.4	515	11.26%	1117	1983.33	6.14	0.01

Sample Code	Small Read Archive Run Accession Number	Number of Seqs. (raw)	Number of Seqs. After Trimming	Average Seq. Length After Trimming	Chimeric Seq.	% Chimeric	OTUs (96% sequence identity)	Chao1 Index	Shannon Index	Simpson Index
HOR-11-65A	SRR628550	11957	7244	331.6	432	5.96%	539	720.22	5.01	0.01
HOR-11-65B	SRR628551	11105	8110	355.5	383	4.72%	1674	2469.89	6.55	0.00
HOR-11-65C	SRR628552	7597	5370	349.3	176	3.28%	1210	1926.09	6.14	0.01
HOR-11-67A	SRR628553	8207	5963	349.3	335	5.62%	2114	3600.72	7.17	0.00
HOR-11-67B	SRR628554	9597	6954	351.5	345	4.96%	2297	3801.25	7.20	0.00
HOR-11-67C	SRR628555	7935	5697	346.8	216	3.79%	1950	3208.50	7.09	0.00
HOR-11-68A	SRR628556	7859	5716	355.1	287	5.02%	2169	3669.43	7.24	0.00
HOR-11-68B	SRR628557	7520	5576	355.2	276	4.95%	2061	3453.50	7.13	0.00
HOR-11-68T	SRR628558	8259	6411	360.1	746	11.64%	502	746.22	4.76	0.02
**TOTAL**		**871175**	**581759**	**40451.97**	**28511**	**4.90%**	-	-	-	-
