# Supplementary material for: Regime Shift in Sandy Beach Microbial Communities following Deepwater Horizon Oil Spill Remediation Efforts
Source: PLoS One. 2014 Jul 18;9(7):e102934. doi: 10.1371/journal.pone.0102934 (PMC4103866; doi:10.1371/journal.pone.0102934)
Supplement: Table S3 — Summary of the number of pyrosequences obtained for each major taxonomic division for sediment samples collected from Grand Isle, Louisiana, 2010–2011. (PDF) [file pone.0102934.s010.pdf]

Table S3. Summary of the number of pyrosequences obtained for each major taxonomic division for sediment samples collected from Grand Isle, Louisiana, 2010-2011.

Sample Number	*Alphaproteobacteria*	*Betaproteobacteria*	*Gammaproteobacteria*	*Deltaproteobacteria*	*Epsilonproteobacteria*	*Acidobacteria*	*Actinobacteria*	*Bacteroidetes*	*Chloroflexi*	*Firmicutes*	*Gemmatimonadetes*	*Nitrospira*	*Planctomycetes*	*Proteobacteria*	*Spirochaetes*	*Verrucomicrobia*	*Cyanobacteria*	*TM7*	*WS3*	*Tenericutes*	*SR1*	*Chrysiogenetes*	*OD1*	*OP10*	*BRC1*	*Fibrobacteres*	*OP11*	*UnclassifiedWS3*	*UnclassifiedTM7*	*UnclassifiedOD1*	*UnclassifiedSR1*	*UnclassifiedOP10*	*UnclassifiedOP11*	*Unclassified-BRC1*	*Total*
HOR-10-18A	73	88	250	4	0	0	14	130	81	546	0	0	0	415	0	2	51	0	0	2	0	0	0	0	0	0	0	0	0	0	0	0	0	0	1369
HOR-10-18B	84	16	38	32	1882	368	406	202	0	2358	2	2	13	2052	3	0	34	0	0	0	0	0	0	0	0	0	0	0	0	0	0	0	0	0	5463
HOR-10-39A	355	774	1113	67	146	82	202	435	19	1161	116	0	12	2455	2	163	88	19	0	1	0	0	0	13	0	0	0	0	19	0	0	13	0	0	4792
HOR-10-39B	374	398	1238	254	55	325	243	702	151	1226	70	56	91	2319	5	96	66	8	3	0	0	3	2	3	0	0	0	3	8	2	0	3	0	0	5462
HOR-11-73A	2402	149	507	64	0	0	1890	171	6	444	0	40	43	3122	0	9	19	111	0	0	0	0	0	0	0	0	0	0	111	0	0	0	0	0	5901
HOR-11-73B	826	187	349	64	5	76	1253	76	50	187	43	9	47	1428	0	53	0	5	0	0	0	0	0	6	0	0	0	0	5	0	0	6	0	0	3235
HOR-10-20A	264	100	2239	261	78	604	539	413	50	2846	107	10	161	2942	0	142	604	0	3	0	2	0	0	0	0	0	0	3	0	0	2	0	0	0	8514
HOR-10-20B	313	15	1394	357	60	782	231	79	230	1190	63	39	277	2139	0	57	23	0	8	0	0	1	0	0	0	0	0	8	0	0	0	0	0	0	5290
HOR-10-42A	285	745	1736	267	17	288	113	248	169	354	29	10	111	3050	5	37	34	3	8	1	0	0	0	0	6	0	0	8	3	0	0	0	0	6	4503
HOR-11-74A	424	143	89	38	0	0	1722	133	11	257	11	0	3	694	0	1	0	31	0	9	0	0	0	0	0	0	0	0	31	0	0	0	0	0	2596
HOR-11-74B	515	188	192	187	4	303	888	70	131	108	122	5	69	1086	16	108	13	141	0	0	0	0	3	0	1	0	0	0	141	3	0	0	0	1	3100
HOR-11-75A	1615	53	3819	46	3	0	147	740	22	112	3	3	89	5536	1	28	326	15	0	0	0	0	0	0	1	0	0	0	15	0	0	0	0	1	7111
HOR-11-75B	876	653	802	653	18	531	204	96	328	209	100	476	209	3002	15	104	17	17	6	0	0	0	0	1	1	0	0	6	17	0	0	1	0	1	5504
HOR-11-76A	2266	25	826	66	5	0	12	1040	12	41	2	1	63	3188	3	136	1936	0	0	0	0	1	0	0	0	0	0	0	0	0	0	0	0	0	6499
HOR-11-76B	1512	274	2458	618	19	667	797	173	177	289	16	33	202	4881	28	19	305	111	12	2	0	2	4	0	0	0	0	12	111	4	0	0	0	0	7946
HOR-11-77A	1158	84	656	240	17	0	604	41	307	1014	0	4	20	2155	3	208	864	0	0	3	0	0	0	0	0	0	0	0	0	0	0	0	0	0	5356
HOR-11-77B	765	45	3301	490	390	150	315	384	167	254	26	13	88	4991	47	24	7	7	4	17	0	0	2	0	0	0	0	4	7	2	0	0	0	0	6608
HOR-11-78A	2981	21	2285	63	4	0	62	686	12	51	1	4	5	5354	0	53	129	0	0	0	0	1	1	0	0	0	0	0	0	1	0	0	0	0	6382
HOR-11-78B	726	70	1816	9	4	403	326	517	182	378	65	29	117	3067	5	34	8	4	2	1	0	0	0	0	0	0	0	2	4	0	0	0	0	0	5236
HOR-11-79A	1213	34	2592	116	0	0	69	1689	52	91	17	4	34	3955	5	12	40	1	0	0	0	0	0	0	0	0	0	0	1	0	0	0	0	0	6057
HOR-11-79B	663	55	1233	472	5	447	222	1089	189	350	69	13	117	2428	4	33	63	1	10	2	0	0	0	0	0	0	0	10	1	0	0	0	0	0	4952

Sample Number	*Alphaproteobacteria*	*Betaproteobacteria*	*Gammaproteobacteria*	*Deltaproteobacteria*	*Epsilonproteobacteria*	*Acidobacteria*	*Actinobacteria*	*Bacteroidetes*	*Chloroflexi*	*Firmicutes*	*Gemmatimonadetes*	*Nitrospira*	*Planctomycetes*	*Proteobacteria*	*Spirochaetes*	*Verrucomicrobia*	*Cyanobacteria*	*TM7*	*WS3*	*Tenericutes*	*SR1*	*Chrysiogenetes*	*OD1*	*OP10*	*BRC1*	*Fibrobacteres*	*OP11*	*UnclassifiedWS3*	*UnclassifiedTM7*	*UnclassifiedOD1*	*UnclassifiedSR1*	*UnclassifiedOP10*	*UnclassifiedOP11*	*Unclassified-BRC1*	*Total*
HOR-10-21B	112	54	498	123	33	265	80	472	145	5694	76	0	111	820	0	5	0	0	10	0	0	0	7	0	0	0	0	10	0	7	0	0	0	0	7816
HOR-10-43A	367	61	1308	299	82	170	55	332	106	2330	9	0	62	2117	13	31	216	0	11	21	0	0	0	0	0	2	0	11	0	0	0	0	0	0	5540
HOR-11-80A	431	4	1886	223	6	163	167	810	96	566	32	4	105	2620	3	46	40	2	6	8	0	0	0	0	0	0	0	6	2	0	0	0	0	0	4712
