# Supplementary material for: Regime Shift in Sandy Beach Microbial Communities following Deepwater Horizon Oil Spill Remediation Efforts
Source: PLoS One. 2014 Jul 18;9(7):e102934. doi: 10.1371/journal.pone.0102934 (PMC4103866; doi:10.1371/journal.pone.0102934)
Supplement: Table S4 — Summary of the number of pyrosequences obtained for each major taxonomic division for sediment samples collected from Dauphin Island, Alabama, 2010–2011. (PDF) [file pone.0102934.s011.pdf]

Table S4. Summary of the number of pyrosequences obtained for each major taxonomic division for sediment samples collected from Dauphin Island, Alabama, 2010-2011.

Sample Number	*Alphaproteobacteria*	*Betaproteobacteria*	*Gammaproteobacteria*	*Deltaproteobacteria*	*Epsilonproteobacteria*	Proteobacteria	Acidobacteria	Actinobacteria	Aquificae	Bacteriodetes	Caldiserica	Chlamydiae	Chlorobi	Chloroflexi	Deferribacteres	Deinococcus-Thermus	Firmicutes	Fusobacteria	Gemmatimonadete	Lentishaerae	Nitrospira	Planctomycetes	Spirochaetes	Synergistales	Thermodesulfobacteria	Thermotogae	Verrucomicrobia	Unclassified Bacteria_incertae_sedis	Cyanobacteria	Candidate Division TM7	Candidate Division WS3	Tenericutes	Candidate Division SR1	Chrysiogenetes	Candidate Division OD1	Candidate Division OP10	Candidate Division BRC1	Fibrobacteres	Candidate Division OP11	Unclassified Candidate Division TM7	Unclassified Candidate Division WS3	Unclassified Candidate Division OD1	Unclassified Candidate Division SR1	Unclassified Candidate Division OP10	Unclassified Candidate Division OP11	Unclassified Candidate Division BRC1	*Total*
HOR-10-03	109	305	936	312	159	1821	2	513	0	283	3	0	0	50	21	0	3403	28	0	0	0	0	0	2	0	1	7	0	48	0	0	20	0	0	0	0	0	0	0	0	0	0	0	0	0	0	6230
HOR-10-06	106	29	650	21	21	827	0	621	0	99	0	0	0	0	0	1	2218	2	0	0	0	0	0	0	0	0	0	0	6	0	0	0	0	0	0	0	0	0	0	0	0	0	0	0	0	0	3787
HOR-10-07	106	18	664	19	650	1457	89	31	0	115	0	0	0	1	0	0	1119	18	0	0	10	4	0	0	0	0	0	0	0	3	0	0	0	0	0	0	0	0	0	3	0	0	0	0	0	0	2847
HOR-10-23A	897	701	243	186	16	2043	689	599	47	643	3	1	0	191	2	9	243	5	148	12	17	78	0	2	4	9	101	5	4	43	0	0	0	5	5	33	8	0	0	43	0	5	0	33	0	8	4949
HOR-10-23B	316	571	173	159	9	1228	599	317	28	486	7	3	2	113	0	4	291	0	70	3	21	78	0	1	6	4	240	1	2	23	0	2	0	0	5	8	1	0	0	23	0	5	0	8	0	1	3543
HOR-10-23C	1104	849	95	106	1	2155	697	645	4	1236	0	0	0	44	0	19	129	4	71	1	3	26	0	1	0	0	5	44	6	4	0	1	0	0	3	13	0	0	0	4	0	3	0	13	0	0	5113
HOR-10-24A	294	405	122	70	6	897	502	237	19	466	1	2	0	118	1	0	119	2	93	2	23	35	0	1	2	0	50	8	1	9	0	0	0	0	4	1	2	0	0	9	0	4	0	1	0	2	2596
HOR-10-24B	1505	671	169	87	34	2466	394	161	13	719	8	3	2	79	7	0	146	0	55	10	5	80	1	1	8	0	37	0	3	4	0	0	0	0	3	0	0	0	0	4	0	3	0	0	0	0	4210
HOR-10-24C	421	1339	596	314	4	2674	526	415	6	310	4	5	6	99	2	4	243	1	55	26	116	261	10	1	298	7	43	1	2	5	10	0	0	0	1		1	1	0	5	10	1	0	5	0	1	5135
HOR-10-25A	409	1918	724	373	6	3430	534	401	10	369	5	13	24	159	6	5	163	0	79	34	70	232	4	2	129	2	121	0	6	2	22	0	0	0	0	6	0	0	0	2	22	0	0	6	0	0	5828
HOR-10-25B	1153	980	119	67	4	2323	1033	429	31	869	0	0	3	104	0	29	166	4	61	0	15	62	0	1	1	0	9	22	36	18	2	0	0	0	0	1	1	0	0	18	2	0	0	1	0	1	5220
HOR-10-25C	68	499	137	55	2	761	116	47	2	110	1	2	2	44	2	1	53	0	26	2	12	52	1	0	36	0	12	0	2	3	1	0	0	0	1	2	1	0	0	3	1	1	0	2	0	1	1292
HOR-10-26A	282	4912	419	221	8	5842	290	146	5	313	1	15	6	110	2	7	206	0	46	11	112	86	4	3	380	1	78	0	12	43	21	1	0	0	2	2	0	0	0	43	21	2	0	2	0	0	7745
HOR-10-26B	802	1268	432	18	0	2520	506	153	2	1841	0	0	0	17	0	21	52	0	30	0	1	20	0	1	0	0	0	3	5	12	0	0	0	0	0	0	0	0	0	12	0	0	0	0	0	0	5184
HOR-10-26C	237	7202	619	155	7	8220	341	225	2	151	0	18	12	82	1	2	101	0	43	25	53	147	3	0	52	4	43	0	1	8	10	0	0	1	0	2	1	0	0	8	10	0	0	2	0	1	9548
HOR-10-27A	492	1105	997	460	47	3101	706	396	14	1017	2	19	7	267	1	5	295	0	77	45	289	296	3	3	100	7	143	0	3	19	40	0	0	0	0	26	3	0	0	19	40	0	0	26	0	3	6845
HOR-10-30B	63	101	177	119	36	496	162	423	0	48	3	1	0	43	0	0	1428	35	17	5	20	9	1	2	2	17	26	0	49	1	17	0	0	0	0	0	0	0	0	1	17	0	0	0	0	0	2805
HOR-10-27B	178	225	63	12	0	478	247	132	1	195	2	0	0	15	5	0	17	0	14	0	5	15	0	0	0	0	7	0	2	1	0	0	0	0	0	0	1	0	0	1	0	0	0	0	0	1	1137
HOR-10-27C	220	100	187	71	2	580	190	140	0	170	1	2	3	23	0	1	24	0	11	4	15	62	0	0	2	0	8	0	4	7	1	0	0	0	0	0	0	0	0	7	1	0	0	0	0	0	1249
HOR-10-28A	1168	54	3342	29	8	4601	10	23	1	769	0	0	0	1	0	0	10	0	0	4	0	19	1	2	0	0	1	1	8	1	1	0	0	0	0	0	0	0	0	1	1	0	0	0	0	0	5455
HOR-10-28B	1143	149	3255	316	12	4875	262	558	8	1085	0	1	1	154	12	11	60	0	14	46	85	337	2	1	2	3	29	1	75	4	10	0	0	0	1	1	0	0	0	4	10	1	0	1	0	0	7639

Sample Number	*Alphaproteobacteria*	*Betaproteobacteria*	*Gammaproteobacteria*	*Deltaproteobacteria*	*Epsilonproteobacteria*	Proteobacteria	Acidobacteria	Actinobacteria	Aquificae	Bacteriodetes	Caldiserica	Chlamydiae	Chlorobi	Chloroflexi	Deferribacteres	Deinococcus-Thermus	Firmicutes	Fusobacteria	Gemmatimonadete	Lentishaerae	Nitrospira
HOR-10-28C	1959	419	565	23	3	2969	3	31	1	1471	0	2	2	25	0	3	60	0	93	11	1
HOR-10-30A	329	578	1437	356	39	2739	506	643	6	191	3	14	9	73	6	19	2617	35	135	15	103
HOR-10-30C	72	58	170	3	37	340	18	30	3	65	0	0	0	17	0	0	2147	0	10	0	4
HOR-10-33A	252	56	1229	152	3	1692	215	212	1	472	0	2	0	56	19	2	4437	0	1	12	15
HOR-10-33B	62	44	1525	0	11	1642	0	552	2	130	0	3	0	0	0	0	2230	0	0	0	0
HOR-10-33C	333	77	962	443	28	1843	637	442	4	261	1	4	2	129	18	9	920	6	63	75	53
HOR-10-34A	264	137	661	49	3	1114	78	412	0	253	0	1	0	11	0	0	1408	0	36	3	0
HOR-10-34B	432	504	1500	337	35	2808	269	232	3	292	0	2	12	74	18	4	443	20	76	32	66
HOR-10-34C	83	108	2296	40	22	2549	46	156	0	131	0	0	1	13	9	1	739	1	10	8	35
HOR-10-53A	1071	1034	156	97	1	2359	272	649	7	931	0	0	0	56	0	33	151	10	146	2	11
HOR-10-53B	112	286	42	70	1	511	579	356	8	47	0	0	0	46	0	3	51	0	38	2	12
HOR-10-53C	173	444	284	154	2	1057	570	141	7	32	2	0	0	56	1	1	51	1	60	0	29
HOR-10-54A	494	366	181	71	2	1114	105	269	3	301	1	0	1	36	2	21	150	6	46	2	10
HOR-10-54B	90	336	69	96	2	593	380	362	5	60	1	1	1	59	0	8	130	2	46	1	19
HOR-10-54C	309	977	470	360	7	2123	600	206	7	148	4	8	3	152	2	7	165	0	130	19	157
HOR-10-48A	1714	947	2561	930	9	6161	613	320	7	443	0	7	27	153	30	8	369	0	189	43	421
HOR-10-48B	676	719	2209	471	49	4124	303	190	1	219	2	1	6	140	33	0	76	8	33	42	90
HOR-10-48C	162	432	1072	241	14	1921	193	49	1	325	2	4	4	46	20	0	42	4	13	18	34
HOR-10-49A	712	375	629	254	8	1978	386	412	14	989	0	0	19	145	10	12	213	1	39	21	80
HOR-10-49B	775	70	1206	595	41	2687	330	226	7	354	0	4	15	243	39	6	155	34	29	85	117
HOR-10-49C	995	108	2152	908	17	4180	485	371	6	1074	0	11	19	520	106	6	313	24	23	141	101
HOR-10-50A	554	79	779	241	5	1658	341	452	4	393	0	6	0	103	12	9	134	0	32	36	49
HOR-10-50B	607	74	2738	143	4	3566	181	189	2	1052	0	5	0	71	7	3	42	2	10	40	39

Sample Number	Planctomycetes	Spirochaetes	Synergistales	Thermodesulfobacteria	Thermotogae	Verrucomicrobia	Unclassified Bacteria_incertae_sedis	Cyanobacteria	Candidate Division TM7	Candidate Division WS3	Tenericutes	Candidate Division SR1	Chrysiogenetes	Candidate Division OD1	Candidate Division OP10	Candidate Division BRC1	Fibrobacteres	Candidate Division OP11	Unclassified Candidate Division TM7	Unclassified Candidate Division WS3	Unclassified Candidate Division OD1	Unclassified Candidate Division SR1	Unclassified Candidate Division OP10	Unclassified Candidate Division OP11	Unclassified Candidate Division BRC1	*Total*
HOR-10-28C	64	4	0	0	0	602	1	3267	5	0	0	0	0	2	1	0	0	0	5	0	2	0	1	0	0	8618
HOR-10-30A	167	4	16	39	4	32	1	90	1	39	3	0	0	3	13	0	0	0	1	39	3	0	13	0	0	7526
HOR-10-30C	22	0	0	4	17	15	0	19	0	0	0	0	0	0	0	0	0	0	0	0	0	0	0	0	0	2711
HOR-10-33A	37	0	9	0	0	10	0	89	0	2	0	0	0	0	0	0	0	0	0	2	0	0	0	0	0	7273
HOR-10-33B	3	2	0	0	0	6	0	28	0	0	0	0	0	0	0	0	0	0	0	0	0	0	0	0	0	4598
HOR-10-33C	336	4	1	1	2	21	0	162	1	26	1	2	2	0	1	11	0	0	1	26	0	2	1	0	11	5038
HOR-10-34A	29	0	0	0	2	0	0	33	0	0	0	0	0	0	0	0	0	0	0	0	0	0	0	0	0	3386
HOR-10-34B	182	9	2	0	2	59	0	59	14	11	0	0	0	1	0	0	0	0	14	11	1	0	0	0	0	4690
HOR-10-34C	22	1	2	0	1	6	2	23	3	2	0	0	0	0	0	0	0	0	3	2	0	0	0	0	0	3761
HOR-10-53A	86	0	0	5	0	23	4	260	22	0	4	0	0	0	23	6	0	0	22	0	0	0	23	0	6	5060
HOR-10-53B	32	0	0	1	0	26	0	579	18	0	0	0	0	0	2	1	0	0	18	0	0	0	2	0	1	1733
HOR-10-53C	39	0	1	2	2	33	0	1	0	4	0	0	0	0	8	1	0	0	0	4	0	0	8	0	1	2099
HOR-10-54A	55	0	0	2	0	33	0	165	6	0	0	0	0	0	1	1	0	0	6	0	0	0	1	0	1	2330
HOR-10-54B	36	0	1	8	2	34	0	1	17	3	0	0	0	0	17	1	0	0	17	3	0	0	17	0	1	1788
HOR-10-54C	155	2	4	16	0	135	0	27	1	30	0	0	0	1	7	2	0	0	1	30	1	0	7	0	2	4111
HOR-10-48A	606	4	5	3	0	143	0	333	22	12	3	0	0	0	33	0	0	0	22	12	0	0	33	0	0	9955
HOR-10-48B	244	5	1	0	1	23	2	132	2	1	2	0	1	3	0	0	0	0	2	1	3	0	0	0	0	5685
HOR-10-48C	199	6	0	1	1	21	0	50	0	6	0	1	0	0	1	0	0	0	0	6	0	1	1	0	0	2692
HOR-10-49A	285	3	1	4	0	50	0	398	18	5	7	1	0	0	1	2	7	0	18	5	0	1	1	0	2	5150
HOR-10-49B	354	9	7	5	3	79	0	89	11	5	4	0	0	1	0	0	0	1	11	5	1	0	0	1	0	4899
HOR-10-49C	787	23	9	7	5	159	1	503	14	14	7	1	1	1	0	1	0	0	14	14	1	1	0	0	1	8985
HOR-10-50A	318	3	2	1	3	16	0	354	5	7	0	0	0	0	0	1	0	0	5	7	0	0	0	0	1	3939
HOR-10-50B	181	3	3	0	0	36	0	1026	1	1	1	0	0	1	0	0	0	0	1	1	1	0	0	0	0	6462

Sample Number	*Alphaproteobacteria*	*Betaproteobacteria*	*Gammaproteobacteria*	*Deltaproteobacteria*	*Epsilonproteobacteria*	Proteobacteria	Acidobacteria	Actinobacteria	Aquificae	Bacteriodetes	Caldiserica	Chlamydiae	Chlorobi	Chloroflexi	Deferribacteres	Deinococcus-Thermus	Firmicutes	Fusobacteria	Gemmatimonadete	Lentishaerae	Nitrospira	Planctomycetes	Spirochaetes	Synergistales	Thermodesulfobacteria	Thermotogae	Verrucomicrobia	Unclassified Bacteria_incertae_sedis	Cyanobacteria	Candidate Division TM7	Candidate Division WS3	Tenericutes	Candidate Division SR1	Chrysiogenetes	Candidate Division OD1	Candidate Division OP10	Candidate Division BRC1	Fibrobacteres	Candidate Division OP11	Unclassified Candidate Division TM7	Unclassified Candidate Division WS3	Unclassified Candidate Division OD1	Unclassified Candidate Division SR1	Unclassified Candidate Division OP10	Unclassified Candidate Division OP11	Unclassified Candidate Division BRC1	*Total*
HOR-10-50C	605	70	1310	327	11	2323	334	308	7	513	0	6	4	151	37	9	106	0	18	49	85	310	4	4	1	2	48	0	463	13	4	0	0	0	1	0	1	0	0	13	4	1	0	0	0	1	4801
HOR-10-51A	579	83	1504	377	13	2556	348	344	8	478	1	3	2	177	32	7	116	0	37	141	79	462	11	2	10	6	48	1	1411	7	10	1	0	0	0	0	0	0	0	7	10	0	0	0	0	0	6298
HOR-10-51B	348	33	668	855	6	1910	200	193	6	269	0	2	5	105	18	5	52	2	15	31	43	166	3	0	2	1	38	0	420	1	2	0	0	0	1	0	0	0	0	1	2	1	0	0	0	0	2830
HOR-10-52A	3958	770	67	315	2	5112	624	1121	1	1581	0	0	1	34	0	25	117	15	111	1	3	44	0	0	0	0	3	21	13	34	0	4	0	0	4	81	0	0	0	34	0	4	0	81	0	0	8650
HOR-11-58A	949	341	183	110	5	1588	331	1321	11	438	1	1	2	89	0	53	210	2	99	3	16	137	1	2	7	0	43	3	76	2	3	0	0	0	0	13	0	0	0	2	3	0	0	13	0	0	4452
HOR-11-58B	1063	386	213	114	15	1791	420	907	36	284	1	2	0	107	0	19	129	0	224	1	9	161	0	1	7	0	35	9	10	28	0	0	0	0	1	1	5	0	0	28	0	1	0	1	0	5	4188
HOR-11-58C	703	304	74	129	15	1225	602	1675	12	332	6	0	0	187	1	57	89	0	270	4	7	130	0	4	4	2	74	8	6	7	0	2	0	0	0	4	1	0	0	7	0	0	0	4	0	1	4709
HOR-11-59A	819	169	117	123	8	1236	327	1031	10	359	0	1	5	90	1	62	134	0	71	4	11	184	1	2	9	4	16	1	79	2	1	0	0	0	0	3	1	0	0	2	1	0	0	3	0	1	3645
HOR-11-59B	597	306	215	308	6	1432	1580	546	16	56	24	2	4	265	5	7	309	0	38	7	29	306	0	0	23	28	20	0	3	5	39	0	0	0	0	4	0	1	0	5	39	0	0	4	0	0	4749
HOR-11-59C	682	1066	323	261	8	2340	1190	567	10	62	6	1	1	225	4	3	369	0	24	2	15	189	1	1	28	11	11	1	2	14	12	5	0	0	2	9	0	9	1	14	12	2	0	9	1	0	5115
HOR-11-60A	1040	158	194	164	3	1559	562	1456	12	338	0	0	0	162	0	16	245	0	30	2	7	128	0	2	8	3	25	0	260	12	3	0	0	0	0	1	0	0	0	12	3	0	0	1	0	0	4831
HOR-11-60B	446	342	379	334	2	1503	1124	1269	15	112	2	1	7	185	1	10	179	1	88	26	60	266	0	1	11	8	77	0	260	5	63	0	0	0	0	2	0	0	0	5	63	0	0	2	0	0	5276
HOR-11-60C	167	61	1243	173	3	1647	645	146	4	43	1	3	4	82	1	0	2045	0	47	22	48	102	0	3	8	0	26	0	22	0	10	0	0	0	0	3	1	1	4	0	10	0	0	3	4	1	4918
HOR-11-62A	512	16	105	19	0	652	0	79	2	204	0	1	0	13	0	19	4046	0	0	6	0	43	0	0	1	0	35	0	335	0	0	8	2	0	0	0	0	0	0	0	0	0	2	0	0	0	5446
HOR-11-62B	179	68	859	439	1128	2673	75	89	2	403	1	5	24	233	48	7	213	16	3	52	28	109	77	0	6	2	17	1	30	3	15	4	0	0	7	0	0	0	0	3	15	7	0	0	0	0	4143
HOR-11-62C	343	88	1276	413	234	2354	101	112	2	887	1	11	71	204	39	10	151	34	7	48	3	106	73	1	5	0	25	0	33	4	10	5	0	1	4	0	0	0	0	4	10	4	0	0	0	0	4302
HOR-11-63A	641	320	226	8	0	1195	2	371	1	435	0	0	0	8	0	4	2059	0	0	6	0	29	0	0	1	0	7	0	129	0	0	1	0	0	0	0	0	0	0	0	0	0	0	0	0	0	4248
HOR-11-63B	137	133	439	297	2465	3471	87	28	0	462	0	3	24	35	39	1	463	0	3	20	17	70	9	0	0	0	14	1	13	0	0	0	3	0	1	0	2	0	0	0	0	1	3	0	0	2	4766
HOR-11-63C	150	34	364	196	2282	3026	53	33	2	260	0	2	7	35	9	4	186	4	1	6	5	56	12	0	1	1	11	0	5	1	0	25	0	0	0	0	1	0	0	1	0	0	0	0	0	1	3746
HOR-11-64A	2091	120	744	68	0	3023	3	227	0	694	0	1	0	12	2	64	3066	0	0	14	0	82	12	3	1	0	196	0	232	0	0	1	3	0	1	0	0	0	0	0	0	1	3	0	0	0	7565
HOR-11-64B	877	16	1691	295	23	2902	72	103	6	1076	0	2	4	41	12	1	153	20	5	20	11	89	6	2	3	0	52	0	15	0	3	11	0	0	2	0	0	0	0	0	3	2	0	0	0	0	4611
HOR-11-64C	631	40	1130	131	3	1935	48	96	5	1696	0	3	4	39	11	0	62	0	6	18	15	72	7	1	0	0	20	0	11	3	4	0	0	0	1	0	0	0	0	3	4	1	0	0	0	0	4057
HOR-11-65A	2526	531	145	131	0	3333	22	583	5	2440	0	0	0	10	0	9	259	0	21	0	9	75	0	0	1	0	2	0	5	11	0	0	0	0	0	0	0	0	0	11	0	0	0	0	0	0	6811
HOR-11-65B	1005	538	2278	364	13	4198	724	278	5	801	1	16	16	95	10	17	176	0	145	56	125	224	2	1	7	1	278	0	0	533	8	0	0	0	4	2	3	0	0	533	8	4	0	2	0	3	7726

Sample Number	*Alphaproteobacteria*	*Betaproteobacteria*	*Gammaproteobacteria*	*Deltaproteobacteria*	*Epsilonproteobacteria*	Proteobacteria	Acidobacteria	Actinobacteria	Aquificae	Bacteriodetes	Caldiserica	Chlamydiae	Chlorobi	Chloroflexi	Deferribacteres	Deinococcus-Thermus	Firmicutes	Fusobacteria	Gemmatimonadete	Lentishaerae	Nitrospira	Planctomycetes	Spirochaetes	Synergistales	Thermodesulfobacteria	Thermotogae	Verrucomicrobia	Unclassified Bacteria_incertae_sedis	Cyanobacteria	Candidate Division TM7	Candidate Division WS3	Tenericutes	Candidate Division SR1	Chrysiogenetes	Candidate Division OD1	Candidate Division OP10	Candidate Division BRC1	Fibrobacteres	Candidate Division OP11	Unclassified Candidate Division TM7	Unclassified Candidate Division WS3	Unclassified Candidate Division OD1	Unclassified Candidate Division SR1	Unclassified Candidate Division OP10	Unclassified Candidate Division OP11	Unclassified Candidate Division BRC1	*Total*
HOR-11-65C	599	274	1321	360	11	2565	466	163	2	1239	1	4	12	56	3	5	115	0	81	29	90	186	0	0	5	0	78	3	5	67	6	1	0	0	0	10	1	0	0	67	6	0	0	10	0	1	5193
HOR-11-67A	688	90	1698	618	12	3106	499	309	6	531	0	9	5	145	52	4	116	1	45	64	135	303	20	1	5	5	61	3	188	3	8	0	0	1	1	0	1	0	0	3	8	1	0	0	0	1	5627
HOR-11-67B	644	86	2196	677	18	3621	445	351	7	685	0	10	9	187	69	13	137	0	55	74	142	368	17	12	5	3	59	1	324	4	4	0	0	2	0	1	0	0	0	4	4	0	0	1	0	0	6605
HOR-11-67C	467	79	1710	715	9	2980	441	280	6	495	3	7	4	155	47	6	133	5	59	80	127	360	16	10	12	3	52	2	186	2	4	0	0	0	2	0	3	0	0	2	4	2	0	0	0	3	5480
HOR-11-68A	557	78	1731	711	20	3097	376	322	6	360	1	11	2	164	59	5	117	5	50	53	142	332	20	5	5	3	61	3	212	0	11	0	0	2	2	0	0	0	0	0	11	2	0	0	0	0	5426
HOR-11-68B	463	77	1771	686	22	3019	359	272	4	455	0	8	5	172	54	7	117	1	62	65	141	330	16	7	3	6	29	1	156	1	7	0	0	0	0	0	1	0	0	1	7	0	0	0	0	1	5298
HOR-11-68T	1126	12	3805	222	122	5287	72	34	2	75	0	0	5	47	7	2	43	0	4	7	0	38	10	4	0	0	2	1	14	1	10	0	0	0	0	0	0	0	0	1	10	0	0	0	0	0	5665
